# Nanoparticle‐Mediated Bubble Suppression During Droplet Solidification for Mechanical Reinforcement

**DOI:** 10.1002/advs.76909

**Published:** 2026-08-05

**Authors:** Runmiao Gao, Xuan Zhang, Mengjie Song, Ronggui Yang, Keke Shao, Jun Shen, Long Zhang, Shuhuai Yao, Yubing Guo, Ruzhu Wang, Christopher Yu Hang Chao

**Affiliations:** ^1^ Department of Energy and Power Engineering School of Mechanical Engineering Beijing Institute of Technology Beijing China; ^2^ Department of Mechanical Engineering The University of Tokyo Tokyo Japan; ^3^ School of Mechanics and Engineering Science Peking University Beijing China; ^4^ Department of Mechanical and Aerospace Engineering The Hong Kong University of Science and Technology Kowloon Hong Kong China; ^5^ School of Medical Technology Beijing Institute of Technology Beijing China; ^6^ Institute of Refrigeration and Cryogenics MOE Engineering Research Center of Solar Power & Refrigeration Shanghai Jiao Tong University Shanghai China; ^7^ Department of Building Environment and Energy Engineering The Hong Kong Polytechnic University Hong Kong China; ^8^ Department of Mechanical Engineering The Hong Kong Polytechnic University Hong Kong China

**Keywords:** 3D printing, droplet solidification, mechanical strength, nanoparticle, trapped air bubble

## Abstract

Droplet‐based 3D printing can fabricate complex structures, but quantitative regulation over droplet solidification and the mechanical performance of printed components remains essential for its load‐bearing and multi‐functional applications. Leveraging the intrinsic transparency of ice, we innovatively propose a nanoparticle‐mediated strategy to suppress trapped air bubbles during water droplet solidification and thereby reinforce the mechanical performance of printed components. We develop a unified influencing factor to integrate the effects of nanoparticle concentration, diameter, and type on droplet nucleation and freezing characteristics. We uncover that the addition of nanoparticles raises nucleation temperature, refines ice dendrites, reduces freezing rate, and ultimately diminishes trapped air bubbles. These effects enable mechanical reinforcement of components and quantitative regulation of their compressive strength. The bubble volume fraction is reduced by ∼35% while the compressive strength is increased by up to 39%, exceeding the reported average values by more than two times. The low‐cost strategy requires no external physical fields and introduces negligible changes to the hydrodynamic properties of raw printing materials. These findings elucidate the physical mechanisms governing nanoparticle‐mediated bubble suppression during droplet solidification and further provide a viable pathway for the controllable fabrication of high‐performance composite printing materials.

## Graphical abstract

Nanoparticles mediate droplet solidification by raising nucleation temperature, refining dendrites, and slowing freezing‐front advance, thereby suppressing trapped air bubbles in droplet‐based 3D printing. This strategy reduces bubble volume fraction by ∼35% and increases compressive strength by up to 39%, offering a low‐cost route to stronger printed components without external fields.

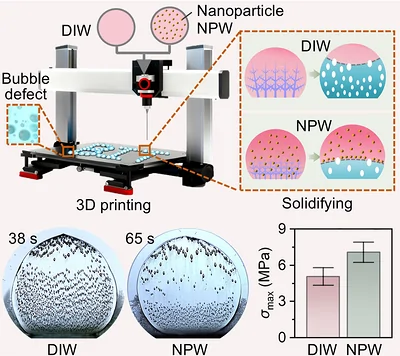

## 1 Introduction

Droplet‐based 3D printing builds 3D structures by ejecting individual droplets of liquid materials only when required [1, 2] and thus has a capability for highly precise and efficient fabrication of complex geometries. It has been widely used in biomedical engineering [3], electronics manufacturing [4], mechanical engineering [5], and customized device manufacturing [6]. However, as droplet‐based 3D printing advances from prototype manufacturing toward the production of load‐bearing and functional components, the mechanical properties of printed components have become a critical bottleneck limiting their engineering deployment [7]. Droplet‐based 3D printing relies on the solidification of liquid droplets, involving nucleation, interface advancement, and heat and mass transfer [8]. The complex solidification process governs microscopic structural evolution and ultimately determines the macroscopic mechanical performance and the reliability of printed components [2, 9]. Therefore, elucidating the physical mechanism of droplet solidification and its controllable regulation is fundamentally crucial not only for reinforcing droplet‐based 3D‐printed components but also for advancing the development of high‐performance composite materials.

To reinforce the mechanical performance of droplet‐based 3D‐printed components, three common strategies have been explored. The first two methods are adjusting printing parameters, e.g., process or structural configurations [10], and imposing external physical fields, e.g., light [11] or magnetic fields [12]. They can partially influence crystal evolution during solidification but are accompanied by increased energy consumption and system complexity. The resulting increase in compressive strength is modest, typically below 20% [13]. The third is introducing reinforcement additives, such as platelets [14] or fibers [15], which can significantly strengthen printed components, but the effectiveness critically depends on precise control over the dispersion and orientation of additives during droplet solidification [14]. Besides, polymer additives often alter ink viscosity and flow behavior, leading to nozzle clogging and other processing issues [16]. In contrast, nanoparticles demonstrate distinctive advantages of homogeneous dispersion in base fluids [17], minimal impact on flowability, and enhanced interfacial bonding [18], making them particularly promising for mechanical reinforcement in droplet‐based 3D printing. However, most existing research on nanoparticle additives focused on the macroscopic mechanical enhancement [18], without elucidating how nanoparticles regulate microscopic droplet solidification behaviors. Specifically, the role of nanoparticles in adjusting the formation and distribution of trapped air bubbles in solidified droplets arising from gas‐liquid solubility differences remains unresolved [19, 20], fundamentally restricting the predictability and reproducibility of mechanical strength regulation in 3D printing [21].

The limited understanding of droplet solidification mechanisms and microscopic defects stems largely from the intrinsically opaque materials in conventional 3D printing [22], which severely hinders in situ observation and quantitative analysis of microscopic structural evolution during printing. Intriguingly, water and its solid phase, ice, offer exceptional optical transparency and a well‐defined solid‐liquid interface [23], providing a unique platform for real‐time visualization of the solidification process. Specifically, the growth and spatial distribution characteristics of trapped air bubbles in ice are first systematically reported by Song et al. [8, 19]. By ingeniously constructing 2D droplets using a Hele‐Shaw cell, they successfully overcome the optical obstruction encountered in 3D droplets, enabling the dynamic capture of trapped air bubbles and temperature distributions [8]. Based on such platforms, various solidification features, including ice dendrite growth [8], freezing front advancement [24], droplet morphology evolution [25], and bubble trapping behaviors [19], can be directly observed. Given the advantages in solidification visualization, water droplets serve as an ideal experimental medium to quantitatively explore the microscopic solidification behaviors of nanoparticle droplets and their relationship with the mechanical performance of 3D‐printed components.

Here, by leveraging droplet‐based 3D printing of ice as an optically transparent model platform to bypass the visualization boundaries of traditionally opaque engineering inks, we propose a nanoparticle‐mediated strategy to suppress trapped air bubbles during water droplet solidification and thereby reinforce the mechanical performance of droplet‐based 3D‐printed components. This strategy may provide mechanistic insights for conventional polymer composites and liquid metal jetting by offering a framework that can be adapted to guide the mitigation of structural porosity. Moreover, this strategy avoids substantial perturbations to fluid flow and heat transfer associated with conventional functional additives and practical constraints when imposing external regulation fields. By constructing 2D droplets in Hele‐Shaw cells, we achieve high‐resolution visualization of the entire solidification process. We develop a unified influencing factor to integrate the effects of nanoparticle concentration, diameter, and type on droplet nucleation and freezing characteristics. We reveal the physical mechanisms of how nanoparticles raise nucleation temperature, refine ice dendrites, and reduce freezing rate. We uncover that denser ice dendrites and a slower freezing rate diminish trapped air bubbles. These mechanisms enable mechanical reinforcement of printed components and quantitative regulation of their compressive strength. The bubble volume fraction is reduced by ∼35% while the compressive strength is increased by up to 39%, exceeding the reported values by more than two times. Beyond advancing the fundamental understanding of bubble formation during solidification, these findings offer a low‐cost, predictable, and quantitative route to mechanical reinforcement in 3D printing, with potential extension to ice fabrication, metal casting, and other materials solidification.

## 2 Results

### 2.1 Droplet‐Based 3D Printing Technology With Nanoparticle Suspensions

Figure 1A illustrates the nanoparticle‐mediated bubble suppression during droplet solidification for increased mechanical strength in droplet‐based 3D printing. Nanoparticle‐suspension water (NPW) droplets are formed by introducing nanoparticles of a specific type, concentration, and diameter into deionized water (DIW) to regulate the droplet solidification process. Compared to DIW droplets, NPW droplets exhibit a higher nucleation temperature and a reduced freezing rate, resulting in fewer bubble defects during droplet solidification and enhancing the mechanical performance of printed components.

**FIGURE 1:**
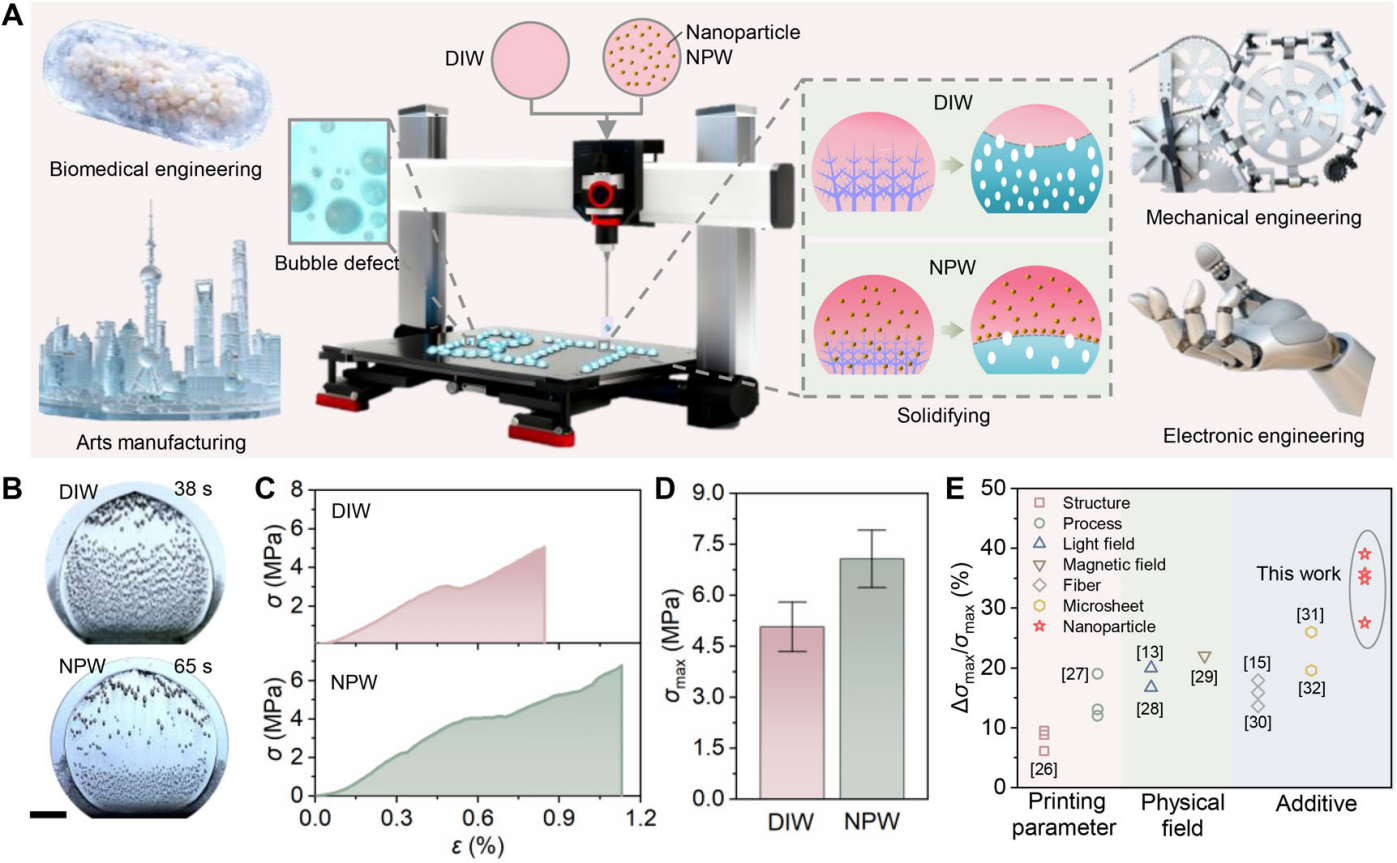
Overview of droplet‐based 3D printing technology with nanoparticle suspensions. (A) Schematic illustration of nanoparticle‐mediated bubble suppression during droplet solidification for enhancing the mechanical strength of printed components. Compared to deionized water (DIW) droplets, nanoparticle water (NPW) droplets exhibit a higher nucleation temperature and a reduced freezing rate, resulting in fewer trapped bubbles during solidification. (B) Experimental images of 2D solidified DIW and TiO_2_‐NPW droplets at *T*_c_ = ‐20°C. NPW droplets contain TiO_2_ nanoparticles with a 20 nm diameter and a 0.01 vol% concentration. The volume of each 2D droplet is 2 µL. The scale bar is 1 mm. The addition of TiO_2_ nanoparticles prolongs the solidification duration of the droplets from 38 s to 65 s, increasing by 71.05%, and reduces the number of trapped air bubbles as the bubble size increases. (C) Variations of stress (*σ*) with strain (*ε*) for solidified 3D DIW and NPW droplets. The addition of nanoparticles increases the maximum strain from 0.85% to 1.13%, representing a 32.94% improvement. (D) Comparisons of maximum compressive stress (*σ*_max_) between 3D solidified DIW and TiO_2_‐NPW droplets. The addition of nanoparticles causes the compressive stress to rise from 5.07 to 7.07 MPa, by 39.45%. (E) Improvement comparison of the maximum compressive strengths achieved by various optimization strategies. The maximum compressive strength of printed components increases by up to 39% in this work, significantly higher than previously reported results.

To investigate the solidification behavior of individual droplets and elucidate the mechanism of nanoparticle‐mediated regulation, 2D droplets prepared by the Hele‐Shaw cell in experiments (Figure S1 and Video S1) enable real‐time observation of the solidification process of DIW and NPW droplets. Based on the time evolution curves of internal temperatures (Figure S2), the four stages, i.e., supercooling, nucleation and recalescence, freezing, and cooling stages, can be observed during the solidification processes of 2D DIW and NPW droplets and 3D ones.

At the freezing stage, the freezing front is advancing with trapped air bubbles forming (Figure S3). The addition of TiO_2_ nanoparticles prolongs the solidification duration from 38 to 65 s, increasing by 71.05%, and reduces the number of trapped air bubbles as their size increases (Figure 1B). In the solidified 2D DIW and TiO_2_‐NPW droplets, with the increase of height, the bubble width, aspect ratio, and volume generally increase while the bubble number basically decreases first and then increases (Figure S3). To assess the mechanical strength of solidified 3D DIW and TiO_2_‐NPW droplets, in situ mechanical compressive experiments are carried out. The stress (*σ*)—strain (*ε*) curves depicted in Figure 1C indicate that the addition of nanoparticles increases the maximum strain from 0.85% to 1.13%, signifying a 32.94% enhancement. Correspondingly, the maximum compressive stress (*σ*_max_) increases from 5.07 to 7.07 MPa, a 39.45% enhancement (Figure 1D). A comparison of the improvements in maximum compressive strength achieved through various optimization strategies is presented in Figure 1E, demonstrating that the addition of nanoparticles yields an increase of 39%, with an average increase of 34%, representing a 2 times improvement compared to the average enhancement efficiency of 16% reported in previous studies [13, 15, 26, 27, 28, 29, 30, 31, 32] and a 31% increase over the reported maximum of 26% [31].

### 2.2 Nucleation and Dendrite Growth

The effect of nanoparticle concentration (*α*), diameter (*D*), and type on droplet nucleation temperature is first investigated by statistical methods (Figure S4). The nucleation temperature normally goes higher with the nanoparticle concentration increasing and the nanoparticle diameter decreasing (Figure 2A–C), a behavior dominated by the Gibbs free energy barrier [33], i.e.,

(1) $$\Delta G^{*}=\frac{16\pi \gamma_{\mathrm{iw}}^{3}T_{f}^{2}f\left(\theta_{Y}\right)}{3\Delta H_{V}^{2}{\left(T_{f}-T_{n}\right)}^{2}}$$

where *γ*_iw_ is the effective interfacial energy between ice and NPW, *T*_f_ and *T*_n_ are the freezing and nucleation temperatures, *f*(*θ*_Y_) is the Fletcher factor related to the Young's contact angle (*θ*_Y_) of the substrate, and Δ*H*_V_ is the volumetric enthalpy of ice melting. In the NPW droplets, nanoparticles substitute part of the water molecules at the ice‐water interface [34] (Figure 2D), and the effective interfacial energy is reduced from *γ*_iw,0_ to *γ*_iw,_ where *γ*_iw,0_ is the interfacial energy between ice and pure water, described by *γ*_iw_ = *γ*_iw,0_ – *γ*_pw_ *f*_p_, where *γ*_pw_ is the interfacial energy between nanoparticles and pure water and *f*_p_ is the area fraction occupied by nanoparticles. Additionally, nanoparticle diameter affects nucleation by altering the molecular arrangement of the ice nucleus, characterized by the function g(D∼)≈[f(θn)−3D∼|cosθn−cosθface|/3D∼|cosθn−cosθface|44]/[f(θn)−3D∼|cosθn−cosθface|/3D∼|cosθn−cosθface|44]f(θface)f(θface) [35]. Here, D∼=Dn/DnDD is the characteristic dimension of a nanoparticle, where *D*_n_ is the critical nucleation radius of the ice nucleus, *θ*_face_ is the angle between the ice‐water interface and the ice‐nanoparticle interface, *θ*_n_ is the apparent contact angle of the ice nucleus, and *f*(*θ*_n_) = 0.5(1‐cos*θ*_n_)‐0.25sin^2^ *θ*_n_cos*θ*_n_ is the volume fraction of the plane‐truncated spherical cap. Consequently, the Gibbs free energy barrier for NPW droplet nucleation is given by

(2) $$\frac{\Delta G^{*}}{\Delta G_{0}^{*}}=g\left(\tilde{D}\right){\tilde{\gamma }}_{iw}^{3}$$

where Δ*G*_0_ ^*^ is the Gibbs free energy barrier for a DIW droplet and ${\tilde{\gamma }}_{iw}^{3}=\gamma_{iw}^{3}/\gamma_{iw,0}^{3}$.

**FIGURE 2:**
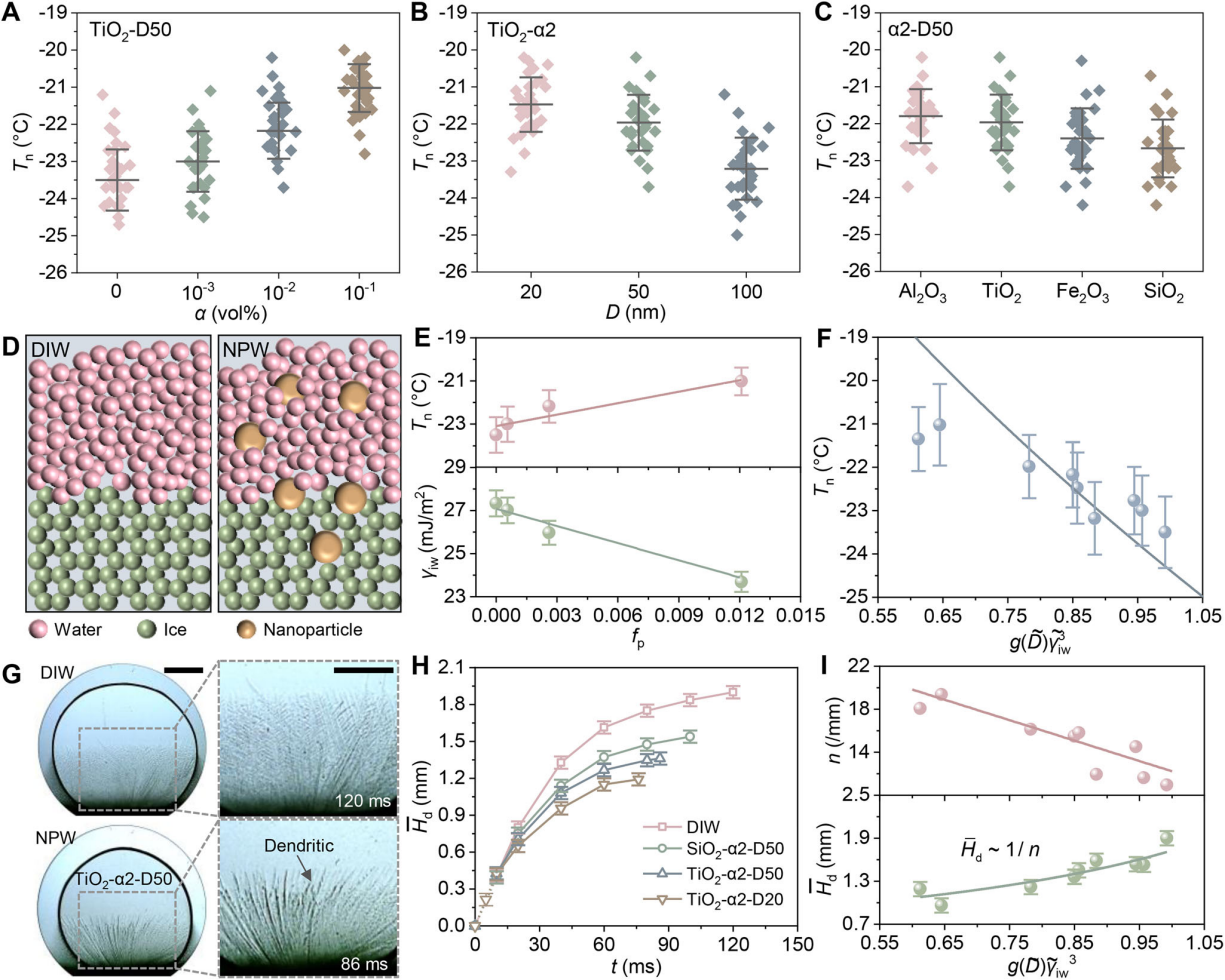
Nucleation temperature and dendritic growth of DIW and NPW droplets. (A–C) Variations of nucleation temperature (*T*_n_) with nanoparticle concentration (*α*), diameter (*D*), and type tested at *V*_droplet_ = 2 µL. TiO_2_‐α2‐D50 denotes an NPW droplet containing TiO_2_ nanoparticles at *D* = 50 nm and *α* = 0.01 vol%. The nucleation temperature exhibits a positive and negative correlation with the nanoparticle concentration and diameter, while Al_2_O_3_‐NPW droplets yield the highest nucleation temperature. (D) Molecular distributions near the ice‐water interface in the DIW and NPW droplets during solidification. Nanoparticles promote heterogeneous nucleation near the ice‐water interface by occupying water molecules. (E) Variation of nucleation temperature and effective ice‐water interfacial energy (*γ*_iw_) with the area fraction (*f*_p_) of nanoparticles. As the area fraction of nanoparticles increases, the nucleation temperature is elevated, and the ice‐water interfacial energy is reduced. (F) Change of nucleation temperature with unified influencing factor [$g\left(\tilde{D}\right){\tilde{\gamma }}_{\mathrm{iw}}^{3}$]. *T*_n_ can be scaled as (Tf−Tn)/(Tf−Tn)(Tf−Tn,0)(Tf−Tn,0)∼g(D∼)γ∼iw3. (G) Dendritic structures in DIW and NPW droplets at the end of the nucleation and recalescence stages at *T*_c_ = −20°C. The scale bars in full and magnified views are 1 and 0.2 mm. The addition of nanoparticles decreases dendrite height and increases its number. (H) Time evolution of the average dendrite height (${\bar{H}}_{d}$) under various nanoparticle concentrations, diameters, and types. ${\bar{H}}_{d}∼{\left(T_{f}-T_{n}\right)}^{1.5}t$ at the early stage while the growth rate gradually slows down at the later stage. The addition of nanoparticles shortens the duration of the recalescence stage. For a TiO_2_‐NPW droplet at *D* = 20 nm and *α* = 0.01 vol%, the duration of the nucleation and recalescence stage is reduced to 76 ms, a significant decrease of 36.67% compared to the duration of 120 ms for a DIW droplet. (I) Plots of dendrite number density (*n*) and average dendrite height as a function of the unified influencing factor. The decrease in the unified influencing factor increases the dendrite number density, and the relation between the average dendrite height and dendrite number density can be scaled as ${\bar{H}}_{d}∼1/n$.

In Figure 2A, a rise in nanoparticle concentration from 0 to 0.1 vol% increases dispersed sites that promote a locally ordered molecular layer with enhanced ice‐like structure [36], raising *f*_p_ and reducing *γ*_iw_. This *γ*_iw_ reduction raises *T*_n_, driving a 10.55% increase in average nucleation temperature from −23.5°C to −21.02°C. In Figure 2B, as the nanoparticle diameter increases from 20 to 100 nm, the average nucleation temperature reduces from −21.47°C to −23.21°C, decreasing by 8.1%. The proximity in size between smaller nanoparticles, such as 20 nm, and the critical ice nucleus [100/(*T*_f_‐*T*_n_)] leads to a decrease in the $g(\tilde{D})$ function, which in turn diminishes the nucleation energy barrier [35, 37]. In Figure 2C, Al_2_O_3_‐NPW and SiO_2_‐NPW droplets yield the highest and lowest average nucleation temperatures of −21.47°C and −23.21°C. This discrepancy is driven by a cross‐scale mechanism: Al_2_O_3_ fosters a tighter microscopic hydrogen‐bond network than SiO_2_ [38], macroscopically manifesting as a smaller intrinsic contact angle (*θ*_int_) (35° vs. 55°) [39] and modulated interfacial tension (*γ*_pw_) (Figure S5). This micro‐macro coupling effectively lowers the ice‐water interfacial energy (*γ*_iw_) and Gibbs free energy barrier (Δ*G* ^*^), thereby stabilizing ice nuclei and elevating *T*_n_.

To quantify the effects of the nanoparticle parameters on the nucleation, the nanoparticle‐water interfacial energy is calculated from Young's equation, i.e., *γ*_pw_ = *γ*_pg_ – *γ*_gw_cos*θ*_int_, where *γ*_pg_ and *γ*_gw_ represent the nanoparticle‐gas and gas‐water interfacial energies. The area fraction is estimated by (Note S1)

(3) $$f_{p}∼\alpha^{2/3}$$

This scaling relation represents a stereological projection assuming a uniform distribution of non‐interacting nanoparticles at the onset of phase change. Then, the ice‐water interfacial energy can be described as

(4) $$\frac{\gamma_{\mathrm{iw}}}{\gamma_{\mathrm{iw},0}}=1-f_{p}\frac{\gamma_{\mathrm{pg}}-\gamma_{\mathrm{gw}}\cos\theta_{int}}{\gamma_{\mathrm{iw},0}}$$

The validity of our calculated ice‐water interfacial energy is verified through statistical analysis of nucleation rates (Note S2). The increasing area fraction and intrinsic contact angle of nanoparticles lower the effective ice‐water interfacial energy and ultimately lead to an elevation in the nucleation temperature (Figure 2E and Figure S6). To account for the additional contribution of nanoparticle diameter on the nucleation energy barrier, $g\left(\tilde{D}\right){\tilde{\gamma }}_{\mathrm{iw}}^{3}$ should be employed to estimate the comprehensive influence of nanoparticles on droplet nucleation, defined as the unified influencing factor, with its values shown in Figure S7. Based on Equations (1) and (2), we relate it to the nucleation temperature, i.e.,

(5) $$\frac{T_{f}-T_{n}}{T_{f}-T_{n,0}}∼\sqrt{g\left(\tilde{D}\right){\tilde{\gamma }}_{\mathrm{iw}}^{3}}$$

where *T*_n,0_ is the nucleation temperature of DIW droplets, with the detailed calculation given in Note S3. Intuitively, this dimensionless descriptor bridges distinct physical scales by integrating geometric compatibility [$g(\tilde{D})$, determined by nanoparticle diameter] and chemical compatibility (${\tilde{\gamma }}_{\mathrm{iw}}^{3}$, governed by concentration and type) into a single metric that indexes the relative reduction in the phase‐change energy barrier. As the unified influencing factor of NPW droplets decreases, the nucleation temperature rises (Figure 2F).

After nucleation, the dendrite starts to grow for both DIW and NPW droplets (Figure 2G, Figure S8, and Video S2), entering the recalescence stage. At the early stage, their growth is predominantly governed by the degree of supercooling, leading to nearly identical growth rates [40], and the average dendrite height basically follows ${\bar{H}}_{d}∼{\left(T_{f}-T_{n}\right)}^{1.5}t$ (Note S4). At the later stage (Figure 2H and Figure S9), the growth rate gradually slows down, and an NPW droplet generates more and shorter dendrites than a DIW droplet, shortening the duration of the recalescence stage. For a TiO_2_‐NPW droplet at *D* = 20 nm and *α* = 0.01 vol%, the duration of the nucleation and recalescence stage is reduced to 76 ms, a significant decrease of 36.67% compared to the duration of 120 ms for a DIW droplet. The divergence in dendritic structures also stems from the decrease in the unified influencing factor $g\left(\tilde{D}\right){\tilde{\gamma }}_{\mathrm{iw}}^{3}$ of NPW. The reduced nucleation energy barrier increases the number density (*n*) of activated nucleation sites for primary dendrites (Figure 2I and Figure S10) [33], thereby restricting their individual growth space. An inverse correlation between the average dendrite height and the dendrite number density is acquired, i.e., ${\bar{H}}_{d}∼1/n$ (Note S5), underscoring the competitive interaction between them. Therefore, the unified influencing factor $g\left(\tilde{D}\right){\tilde{\gamma }}_{\mathrm{iw}}^{3}$ of NPW droplets, related to nanoparticle concentration (*α*), diameter (*D*), and type (characterized by intrinsic contact angle (*θ*_int_), modulates both the nucleation temperature and dendritic growth kinetics at the nucleation and recalescence stage.

### 2.3 Freezing Stage and Trapped Air Bubbles

At the freezing stage of both DIW and NPW droplets (Figures S11–S13 and Video S3), air bubbles squeezed out of water are trapped by the advancing freezing front as shown in Figure 3A.

**FIGURE 3:**
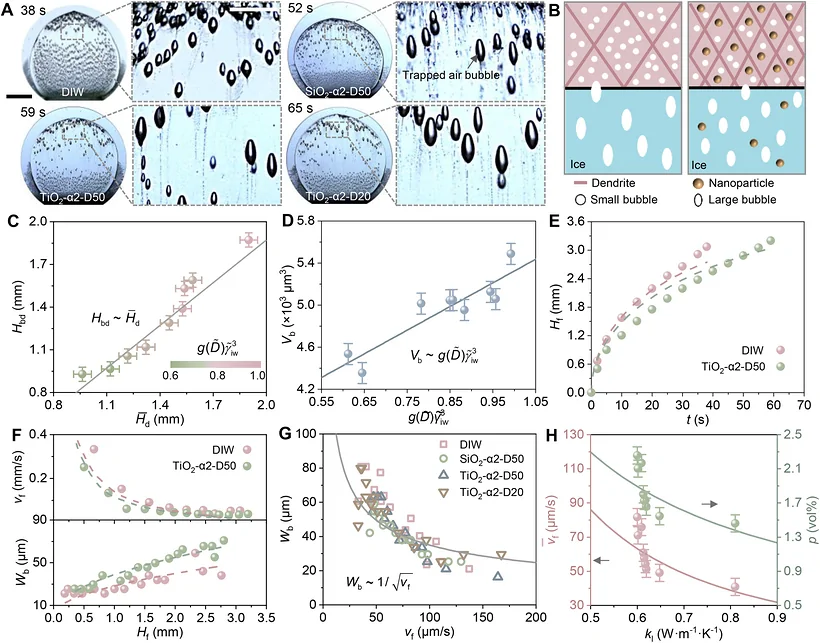
Freezing stage of DIW and NPW droplets and trapped air bubbles in solidified droplets. (A) Experimental images of trapped air bubbles in solidified DIW and NPW droplets at *V*_droplet_ = 2 µL and *T*_c_ = ‐20°C. The scale bars in full and magnified views are 1 and 0.2 mm. The NPW droplets generate larger but fewer bubbles, with a bubble‐dense region found next to the cold plates. (B) Schematic illustration of the formation mechanism of trapped air bubbles near the freezing front in DIW and NPW droplets. In the bubble‐dense region, the addition of nanoparticles densifies the dendritic network in the droplet, with the pore and trapped gas becoming smaller and fewer, resulting in more but smaller trapped air bubbles during freezing. (C) Height of bubble‐dense region (*H*_bd_) vs. the average dendrite height. The height of the bubble‐dense region is nearly linear to the average dendrite height, i.e., $H_{\mathrm{bd}}∼{\bar{H}}_{d}$. (D) Change of average volume of a single bubble (*V*_b_) in the bubble‐dense region with unified influencing factor. The relation between *V*_b_ and $g\left(\tilde{D}\right){\tilde{\gamma }}_{\mathrm{iw}}^{3}$ can be scaled as *V*_b_ ∼ $g\left(\tilde{D}\right){\tilde{\gamma }}_{\mathrm{iw}}^{3}$. (E) Time evolution of the freezing front height (*H*_f_) under various nanoparticle concentrations, diameters, and types. The growth rate of the freezing front height gradually decreases over time. The addition of nanoparticles prolongs the duration of the freezing stage. For a TiO_2_‐NPW droplet at *D* = 50 nm and *α* = 0.01 vol%, the duration of the freezing stage is extended to 59 s, a significant increase of 55.26% compared to the duration of 38 s for a DIW droplet. (F) Variation of the freezing rate (*v*_f_) of droplets and the bubble width with the freezing front height. The freezing rate gradually decreases with the advancement of the freezing front, which in turn leads to an increase in the bubble width. The addition of nanoparticles decreases the freezing rate and increases the bubble width. (G) Change of bubble width with the freezing rate of droplets. The relation between *W*_b_ and *v*_f_ can be scaled as $W_{b}∼v_{f}^{-0.5}$. The nanoparticle‐induced changes in bubble size are attributable to alterations in the freezing rate. (H) Plots of average freezing rate and bubble volume fraction as a function of thermal conductivity (*k*_l_) of liquid droplet. As the thermal conductivity increases from 0.6 W/(m·K) to 0.81 W/(m·K), the average freezing rate reduces from 81.71 to 48.88 µm/s, by 40.18%, leading to a reduction in the bubble volume fraction from 2.25% to 1.46%, by 35.11%.

Concurrently, the macroscopic shape and dynamic deformation of these freezing droplets undergo distinct geometric transitions. These transient morphological profiles across clear nanoparticle concentration gradients are extracted in Figure S14, with the corresponding physical model derivations of the apex singularity and localized apical photographs provided in Note S6. Regardless of nanoparticle concentration, diameter, and type, the trapped air bubbles in the solidified NPW droplets are larger but fewer than those in the DIW ones. Near the cold plate, bubble‐dense regions where the bubbles are much closer to each other and smaller than other regions are observed in all solidified droplets, with the region height defined as *H*_bd_. These tiny bubbles are trapped in the pores of the dendritic network formed at the recalescence stage. As seen in Figure 3B, the addition of nanoparticles densifies the dendritic network and reduces the pore size and dendritic height, resulting in more but smaller trapped bubbles in the bubble‐dense regions as well as a smaller region height. The height of the bubble‐dense region is nearly linear to the average dendrite height, i.e., $H_{\mathrm{bd}}∼{\bar{H}}_{d}$ (Figure 3C), corroborating the spatial confinement of trapped air bubbles by the dendritic structure. Besides, the average volume (*V*_b_) of a single bubble in the bubble‐dense region exhibits an approximately positive linear correlation with the unified influencing factor, i.e., *V*_b_ ∼ $g\left(\tilde{D}\right){\tilde{\gamma }}_{\mathrm{iw}}^{3}$ (Figure 3D). This relationship arises because nanoparticles, by decreasing $g\left(\tilde{D}\right){\tilde{\gamma }}_{\mathrm{iw}}^{3}$, refine the dendritic network, thereby restricting bubble growth space and resulting in smaller trapped air bubbles. When $g\left(\tilde{D}\right){\tilde{\gamma }}_{\mathrm{iw}}^{3}$ = 1, corresponding to DIW droplets, the average bubble volume in the bubble‐dense region is 5887.97 µm^3^. When the $g\left(\tilde{D}\right){\tilde{\gamma }}_{\mathrm{iw}}^{3}$ decreases to 0.65, corresponding to NPW droplets containing TiO_2_ nanoparticles at *D* = 50 nm and *α* = 0.1 vol%, the bubble volume decreases to 4,353.95 µm^3^, a reduction of 26.05%.

Inside the confined dendritic network, bubble growth is constrained by its pore size. Outside this region, trapped bubbles can grow freely, with their growth strongly coupled to the droplet freezing process, especially the advancement of the freezing front [8]. While this 2D simplification successfully bypasses severe optical bottlenecks to achieve high‐resolution in situ visualization, its profound thermodynamic and kinetic correlations with real 3D droplet systems are justified in Note S7. Based on energy conservation, the freezing processes of both 2D DIW and NPW droplets in a Hele‐Shaw cell can be predicted by a model (Note S8), i.e.,

(6) $$\rho_{l}aLdH_{f}=ak_{\mathrm{ice}}\frac{T_{f}-T_{c}}{H_{f}}dt-2ah_{\mathrm{air}}H_{f}\left(T_{\mathrm{air}}-T_{\mathrm{cell}}\right)dt-ah_{l}\left(T_{l}-T_{f}\right)dt$$

where *a* is the gap width of the Hele‐Shaw cell, *H*_f_ is the central height of freezing front, *k*_ice_ is the thermal conductivity coefficient of ice, *L* is the latent heat of solidification, *T*_air_, *T*_cell_ and *T*_l_ are the air temperature, cell temperature and the average temperature of the liquid droplet during freezing (Note S9), *h*_air_ and *h*_l_ are the heat transfer coefficients between the cell and air and between the liquid droplet and freezing front. For the laminar natural convection over a downward‐cooling surface between the liquid droplet and freezing front, *h*_l_ is given by the empirical correlation as *h*_l_ = 0.54*k*_l_ *Gr* ^0.25^ *Pr* ^0.25^/*d* [41], where *Gr* is the Grashof number, *Pr* is the Prandtl number, *d* is the characteristic dimension, *k*_l_ is the thermal conductivity coefficient of the liquid droplet, and *k*_l_ is related to nanoparticle concentration, diameter, and type. It can be expressed as

(7) $$k_{l}∼\left(1+\alpha D^{-3.5}\right)\left[\frac{k_{p}+2k_{water}+2\alpha D^{-3}\left(k_{p}-k_{water}\right)}{k_{p}+2k_{water}-\alpha D^{-3}\left(k_{p}-k_{water}\right)}\right]k_{water}$$

where *k*_water_ and *k*_p_ are the thermal conductivity of water and nanoparticles, with the detailed derivation provided in Note S10. Formally, because *h*_l_ scales proportionally with *k*_l_, an elevated thermal conductivity magnifies the opposing liquid heat flux term in Equation (6). This enhanced heat input directly counteracts part of the ice‐side conductive cooling, slowing down the local freezing front advancement (d*H*_f_/d*t*).

Comparisons with the experimental data in Figure 3E and Figure S15 demonstrate that Equation (6) can predict the time evolution of the freezing front height under various nanoparticle concentrations, diameters, and types. The results also indicate that the growth rate of the freezing front gradually decreases over time (Figure 3E and Figure S16), while the addition of nanoparticles prolongs the duration of the freezing stage. This is attributed to the fact that nanoparticles increase the thermal conductivity of the liquid droplet, which increases the effective liquid‐side heat transfer coefficient between the liquid and freezing front. Thereby, the enhanced heat input from the liquid to the freezing front partially offsets ice‐side cooling, slowing front advancement. For a TiO_2_‐NPW droplet at *D* = 50 nm and *α* = 0.01 vol%, the duration of the freezing stage is extended to 59 s, a significant increase of 55.26% compared to the duration of 38 s for a DIW droplet.

Figure 3F also suggests that as the freezing front advances, the bubble width (*W*_b_) increases owing to the decreasing freezing rate. The addition of nanoparticles reduces the freezing rate and thus increases the width, length, and equivalent diameter of trapped air bubbles (Figure S17). To deconstruct the underlying physics of this bubble formation and growth dynamics itself, the transient absolute and normalized growth histories over time, as well as the evolution of the spatial aspect ratio (*L*_b_/*W*_b_) along the droplet height, are systematically tracked and analyzed (Figures S18 and S19). Figure 3G further depicts the change of bubble width with the freezing rate under various conditions. Interestingly, the experimental data for all nanoparticle concentrations, diameters, and types coalesce into one curve, and the relationship between the bubble width and the freezing rate can be scaled as

(8) $$W_{b}∼v_{f}^{-0.5}$$

which is established based on gas diffusion dynamics (Note S11). This universal data collapse demonstrates that, within the tested parameter range, the scaling is mainly governed by water‐air mass transport, while nanoparticles affect bubble size primarily through *v*_f_.

Following this idea, we further relate the total bubble volume to the freezing rate by (Note S12)

(9) $$\frac{d\sum V_{b}}{dt}\approx 2aC_{\mathrm{air}}{\left(1+\frac{1}{\varsigma c_{air}}\right)}^{-1}\sqrt{R_{0}^{2}-{\left(H_{f}+R_{0}\cos\theta_{\mathrm{droplet}}\right)}^{2}}\frac{dH_{f}}{dt}$$

where *ς* is the ratio of ice density to water density, *θ*_droplet_ is the apparent contact angle of 2D DIW and NPW droplets, *c*_air_ is the air solubility in liquid droplet, *C*_air_ is the correction factor, and *R*_0_ is the initial droplet radius. Combined with Equation (6), integration of Equation (9) yields the time evolution of total bubble volume inside the solidified part. The addition of nanoparticles increases the thermal conductivity in the liquid droplet, leading to a decrease in freezing rate and a corresponding reduction in the bubble volume fraction after solidification, defined as *p* = ∑*V*_b_/(∑*V*_b_+*V*_droplet_/*ς*), as experimental results in Figure 3H confirmed. Specifically, as the thermal conductivity increases from 0.6 W/(m·K) (DIW) to 0.81 W/(m·K) (TiO_2_‐α1‐D50), the average freezing rate (${\bar{v}}_{f}$) reduces from 81.71 µm/s to 48.88 µm/s, by 40.18%, leading to a reduction in the bubble volume fraction from 2.25% to 1.46%, by 35.11%. The above results elucidate the dual mechanism through which nanoparticles affect trapped bubbles during solidification: one is to densify the dendritic network at the recalescence stage by decreasing the unified influencing factor, and the other is to reduce the freezing rate at the freezing stage by enhancing the thermal conductivity of the liquid droplet.

### 2.4 Mechanical Strengths of Solidified Droplets

To further investigate the nanoparticles’ impact on the macroscopic mechanical properties of the solidified droplets, we conduct compressive experiments on single 3D solidified droplets. We develop a mechanical testing setup for solidified droplets, performing in‐situ strength measurements immediately following solidification on the cold plate. The stress—strain curves in Figure 4A and Figure S20 show that the solidified NPW droplets generate higher peak stress than the DIW one, indicating an improvement in the mechanical strength of solidified droplets. For a TiO_2_‐NPW droplet with *α* = 0.01 vol% and *D* = 50 nm, the maximum strain is increased to 1.01%, a significant increase of 18.82% compared to the value of 0.85% for a DIW droplet. The improvement in mechanical strength also depends on the nanoparticle parameters, including concentration, diameter, and type (Figure S21). The results of compressive stresses (Figure 4B) demonstrate that the maximum compressive stress exhibits a positive and negative correlation with the nanoparticle concentration and diameter but is less affected by the nanoparticle type. As the nanoparticle concentration increases from 0 to 0.1 vol%, the maximum compressive stress rises from 5.07 to 6.11 MPa, by 20.51%. As the nanoparticle diameter increases from 20 to 100 nm, the maximum compressive stress reduces from 7.07 to 5.77 MPa, by 18.39%.

**FIGURE 4:**
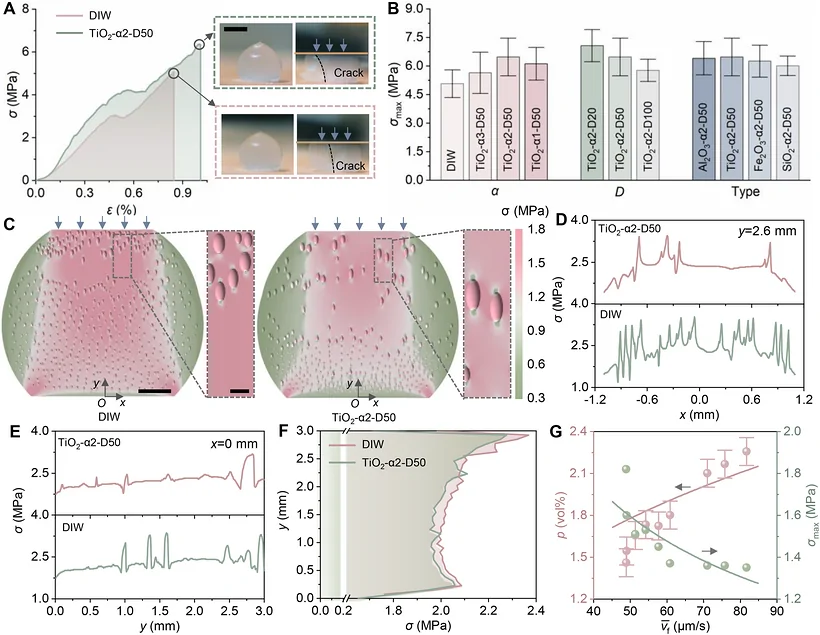
Mechanical strengths of solidified DIW and NPW droplets. (A) Stress—strain curve of solidified 3D DIW and NPW droplets at *V*_droplet_ = 10 µL and *T*_c_ = −20°C. The scale bar is 1 mm. The addition of nanoparticles increases the maximum compressive force. For a TiO_2_‐NPW droplet at *D* = 50 nm and *α* = 0.01 vol%, the maximum strain is increased to 1.01%, a significant increase of 18.82% compared to the maximum strain of 0.85% for a DIW droplet. (B) Variations of maximum compressive stresses with nanoparticle concentration, diameter, and type. The maximum compressive stress exhibits a positive and negative correlation with the nanoparticle concentration and diameter, while TiO_2_‐NPW droplets yield the highest maximum compressive stress. (C) Comparison of equivalent stress distribution contour map between 2D solidified DIW and TiO_2_‐NPW droplets obtained from simulations. The scale bars in full and magnified views are 0.5 and 0.2 mm. A constant force of *F* = 1 N is applied to the sample. A Cartesian coordinate system is established with the origin at the bottom center of the droplet, +*x* to the right and +*y* upward. The TiO_2_‐NPW droplet yields a smaller high‐stress region. (D) Variation of equivalent stress distribution in the *x*‐axis direction at *y* = 2.6 mm. Trapped air bubbles cause stress concentration and fluctuation. (E) Variation of equivalent stress distribution in the *y*‐axis direction at *x* = 0 mm. Trapped air bubbles cause stress concentration and fluctuation. (F) Variation of *x*‐averaged equivalent stress in the *y*‐axis direction. The average values of the curves for solidified 2D DIW and TiO_2_‐α2‐D50 droplets are 2.06 MPa and 1.98 MPa, respectively. (G) Plots of bubble volume fraction and maximum compressive strength of solidified 2D DIW and NPW droplets as a function of average freezing rate. The compressive strengths of the droplets are all calculated from simulations with a permissible tensile strength of *σ*_t_ = 1 MPa. As the average freezing rate decreases from 81.71 to 48.88 µm/s, the bubble volume fraction reduces from 2.25% to 1.46%, by 35.11%, leading to a rise in the compressive strength from 1.35 to 1.82 MPa, by 34.81%.

To reveal the physical mechanism by which the nanoparticles improve the mechanical strength of solidified droplets, we perform simulations to obtain the stress distributions inside the solidified 2D DIW and NPW droplets, as presented in Figure 4C and Figure S22, with detailed information provided in Note S13. Regions of high tensile stresses are developed along the longitudinal axis, aligning well with the crack propagation path observed in Figure 4A. Compared to the DIW droplet, the TiO_2_‐NPW droplet yields a smaller high‐stress region owing to fewer bubble‐induced defects and stress concentration sites [42]. Trapped air bubbles cause stress concentration and fluctuation in both the *x*‐axis and *y*‐axis directions (Figure 4D,E). The stress distribution curve along the *y*‐axis shows a smoother trend with fewer peaks, suggesting that a crack caused by stress perturbations is more likely to appear in the load‐oriented direction. As plotted in Figure 4F, the solidified DIW droplet and TiO_2_‐NPW droplet with *α* = 0.01 vol% and *D* = 20 nm yield the maximum and minimum global average stresses of 2.06 and 1.98 MPa.

Based on the failure criterion of the maximum tensile strength of the solidified droplet being *σ*_t_ = 1 MPa, the maximum compressive stress of solidified droplets under all conditions can be obtained through mechanical simulations, with the detailed derivation provided in Note S13. Following Equation (9) and Figure 3H, we plot the bubble volume fraction and the maximum compressive stress as a function of the freezing rate in Figure 4G, and the relation between the maximum compressive stress and freezing rate approximately satisfies *σ*_max_ ∼ ${{\bar{v}}_{f}}^{-0.4}$. Specifically, as the freezing rate decreases from 81.71 µm/s (DIW) to 48.88 µm/s (TiO_2_‐NPW with *α* = 0.1 vol% and *D* = 50 nm), the bubble volume fraction reduces from 2.25% to 1.46%, by 35.11%, leading to a rise in the compressive strength from 1.35 to 1.82 MPa, by 34.81%. Crucially, although a reduced freezing rate allows individual bubbles to grow larger, the drop in their absolute number drives a net decrease in the total volume fraction. This overall void reduction fundamentally increases the maximum compressive stress by maximizing the effective continuous load‐bearing area and eliminating critical stress‐concentration sites to suppress micro‐crack initiation.

### 2.5 Droplet‐Based 3D Printing Test With Nanoparticle Suspensions

After understanding the underlying physics in regulating the solidification of NPW droplets, we implement this strategy in droplet‐based 3D printing to tune the mechanical performance of printed components via nanoparticle concentration, diameter, and type (Figure 5A). Targeting the practical requirements, we choose five metrics to comprehensively evaluate the 3D printing technology, including four of the raw nanoparticle suspensions, i.e., unified influencing factor [$g\left(\tilde{D}\right){\tilde{\gamma }}_{\mathrm{iw}}^{3}$], thermal conductivity (*k*_l_), viscosity (*µ*), and cost (*c*), and one of the solidified droplets, i.e., maximum compressive strength (*σ*_max_), with detailed information provided in Note S14. For a standardized comparison between 0 and 1, the values of each metric are normalized by

(10) $$\bar{M}=\frac{M_{\max}-M}{M_{\max}-M_{\min}}$$

where *M*_max_, *M*, and *M*_min_ are the maximum, actual, and minimum values. The results show that the TiO_2_‐NPW suspension with *α* = 0.01 vol% and *D* = 50 nm achieves the optimal balance among competing factors (Figure 5B). Comparison with other common nanoparticles, such as graphene (GPE), carbon nanotubes (CNTs), and SiC, demonstrates that SiO_2_, TiO_2_, and Al_2_O_3_ nanoparticles with *α* = 0.01 vol% and *D* = 50 nm exhibit superior comprehensive performance, rendering them more suitable for practical applications in droplet‐based 3D printing (Figure 5C).

**FIGURE 5:**
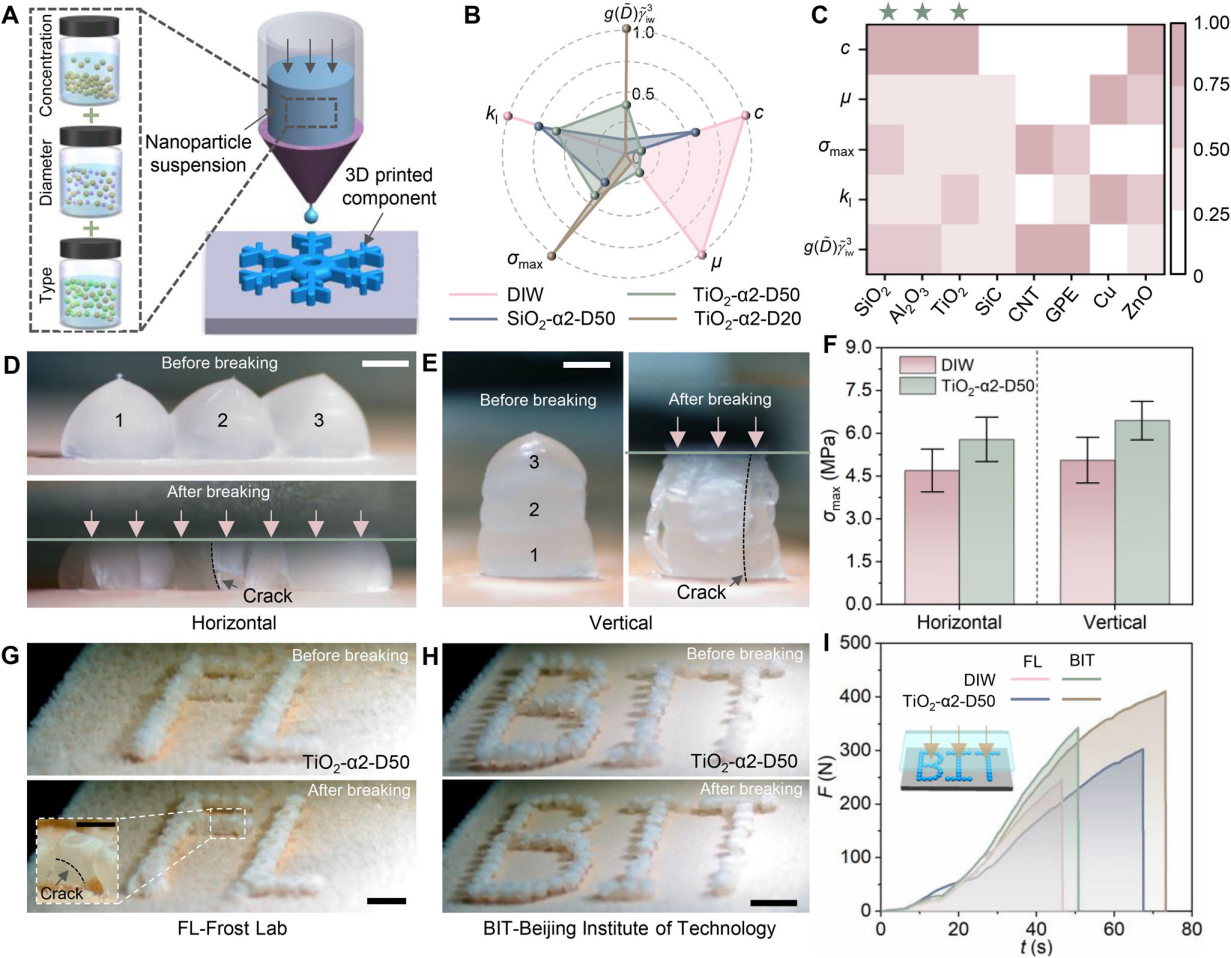
Tests of droplet‐based 3D printing technology with nanoparticle suspensions. (A) Schematic diagram of droplet‐based 3D printing with suspensions containing nanoparticles of various nanoparticle concentrations, diameters, and types. (B) A radar map for a comprehensive performance evaluation of droplet‐based 3D printing. The evaluation criteria include the unified influencing factor [$g\left(\tilde{D}\right){\tilde{\gamma }}_{\mathrm{iw}}^{3}$], thermal conductivity (*k*_l_), viscosity (*µ*), and cost (*c*) of the suspension, as well as the compressive strength (*σ*_max_) of printed components. All the criterion values are normalized to a 0–1 scale with 1 and 0 representing the best and poorest performance. (C) A matrix map evaluating the performance of droplet‐based 3D printing with common nanoparticle suspensions. SiO_2_, TiO_2_, and Al_2_O_3_ nanoparticles are the top choices for superior comprehensive performance. (D,E) Experimental images of mechanical compression tests of multiple formed DIW and NPW droplets stacked horizontally and vertically. The scale bar is 1 mm. (F) Compressive strengths of multiple formed DIW and NPW droplets stacked horizontally and vertically. The addition of nanoparticles increases the maximum compressive strength of horizontally and vertically formed droplets from 4.69 to 5.78 MPa and from 5.05 to 6.44 MPa, by 23.24% and 27.52%. (G,H) Experimental images of mechanical compression tests of printed FL and BIT patterns with DIW and NPW droplets. The scale bars in full and magnified views are 5 and 1 mm. (I) Time evolution of applied downward compressive force on printed FL and BIT patterns. The addition of nanoparticles increases the maximum compressive force of the FL and BIT patterns from 246 to 342 N and from 302 to 410 N, by 39.02% and 35.76%.

With the optimized nanoparticle suspension parameters, i.e., TiO_2_‐NPW with *α* = 0.01 vol% and *D* = 50 nm, we complete the 3D printing of three DIW and NPW droplets stacked horizontally (Figure 5D, Figure S23, and Video S4) and vertically (Figure 5E, Figure S24, and Video S5) and test their compressive strengths. Compared to DIW droplets, the addition of nanoparticles increases the maximum compressive strengths of horizontally and vertically formed droplets from 4.69 to 5.78 MPa and from 5.05 to 6.44 MPa, by 23.24% and 27.52% (Figure 5F). Furthermore, we formed 3D patterns of “FL” (Frost Lab) and “BIT” (Beijing Institute of Technology) by DIW and NPW droplets (Figure 5G,H, Figure S25, and Video S6), followed by compressive tests. The results in Figure 5I show that the addition of nanoparticles increases the maximum compressive force of the FL and BIT patterns from 246 to 342 N and from 302 to 410 N, by 39.02% and 35.76%. The above results from single droplets and linear stacks to complex configurations validate the enhancement effect of nanoparticles on the mechanical strength of printed components across all scales.

#### 2.5.1 Discussion

In summary, leveraging the intrinsic transparency of ice, we innovatively propose a nanoparticle‐mediated strategy to suppress trapped air bubbles during water droplet solidification and thereby increase the mechanical strength of droplet‐based 3D‐printed components and resolve the corresponding fundamental theoretical and technical issues. First, we innovatively develop a unified influencing factor to integrate the effects of nanoparticle concentration, diameter, and type on droplet nucleation and freezing characteristics, providing a general framework for overcoming the challenge of controllable droplet solidification. The nucleation temperature is related to a 0.5‐power law of the unified influencing factor. The decrease in the unified influencing factor increases the dendrite number density and reduces the average dendrite height. Then, we reveal the physical mechanisms of how nanoparticles modulate the freezing rate of droplets. Surprisingly, the addition of nanoparticles lowers the freezing rate, as nanoparticles increase the thermal conductivity of the liquid. We find that the nanoparticle‐induced changes in bubble size are attributed to alterations in the freezing rate, and the bubble width is scaled as a –0.5‐power law of the freezing rate. After confirming the dominant influence of the thermal conductivity of nanoparticle suspension on the freezing rate, we also develop a theoretical model to calculate bubble volume fraction in solidified droplets and achieve a maximum decrease of ∼35% in bubble volume fraction for nanoparticle droplets. By mechanical experiments and simulations, we confirm that the nanoparticle‐induced decrease in the bubble volume fraction of solidified droplets can increase the compressive strength of these droplets. Finally, we apply our regulation strategy to droplet‐based 3D printing. After conducting a comprehensive comparison of the unified influencing factors, thermal conductivity, viscosity, and economic efficiency of common nanoparticle suspensions, and the maximum compressive strength of printed components, we recommend adding SiO_2_, TiO_2_, and Al_2_O_3_ nanoparticles in the raw printing materials. The maximum compressive strength of printed components is increased by up to 39%, exceeding previously reported values by more than 2 times [13, 15, 26, 27, 28, 29, 30, 31, 32]. The low‐cost strategy requires no external physical fields and introduces negligible changes to the hydrodynamic properties of raw printing materials.

To contextualize these findings within the broader landscape of advanced solidification processing, it is instructive to differentiate our droplet‐based configuration from established directional freezing methodologies, such as freeze‐casting and ice‐templating. Physically, our system mirrors freeze‐casting in its multi‐phase boundary transport, where the advancing freezing front induces localized particle segregation and close packing into interstitial domains. However, while conventional freeze‐casting operates as a continuous, macroscopic casting technique to shape and consolidate bulk bodies from liquid slurries, our work targets the precise, individual control of single‐droplet boundary kinetics and transient freezing rates within layer‐by‐layer additive manufacturing sequences. Furthermore, unlike ice‐templating, which deliberately exploits directional ice crystal growth as a temporary, sacrificial scaffold to yield anisotropic, highly porous networks upon sublimation, our strategy pursues a completely inverted structural objective. Instead of using ice as a fugitive pore‐former, we leverage nanoparticle‐mediated thermodynamics to actively suppress microscale trapped air bubbles. This eliminates the need for any post‐processing template removal and directly synthesizes denser, macroscopically reinforced structural components optimized for functional load‐bearing applications.

These findings elucidate the physical mechanisms governing nanoparticle‐mediated bubble suppression during droplet solidification, and the nanoparticle‐mediated bubble suppression increases the mechanical strength of printed components and overcomes the longstanding challenge of simultaneously promoting nucleation, optimizing dendritic structures, controlling solidification kinetics, and inhibiting bubble defects in traditional 3D printing technologies. It also offers new insights into the design of advanced additive manufacturing technologies and high‐performance composite materials, as well as the optimization of the relevant applications, such as phase change energy storage [43]. In this work, we primarily focus on the solidification of NPW droplets. Beyond serving as a transparent model platform, this regulating strategy provides a mechanistic framework for diverse polymer‐based, hydrogel, and high‐performance composite inks, where low‐concentration nanoparticles can similarly optimize phase‐change or curing kinetics to eliminate bubble defects and reinforce macroscopic structural reliability. However, a critical concentration upper limit must be carefully managed in these systems, beyond which dominant van der Waals attractive forces inevitably trigger severe particle aggregation and gravitational sedimentation that compromise ink homogeneity. The particle aggregation within solidified materials and how it affects the mechanical properties remains to be elucidated [44].

## 3 Methods

### 3.1 Preparations of Nanoparticle Suspension

The nanoparticle‐water (NPW) suspensions used in this study are colloidal suspensions formed by dispersing γ‐phase Al_2_O_3_ nanoparticles (50 nm diameter, 99.99% purity), anatase TiO_2_ nanoparticles (20, 50, and 100 nm diameters, 99.95% purity), α‐phase Fe_2_O_3_ nanoparticles (50 nm diameter, 99.95% purity), and hydrophilic SiO_2_ nanoparticles (50 nm diameter, 99.95% purity) in deionized water (DIW). These specific oxides are strategically selected to establish a systematic gradient of both intrinsic contact angles (35° to 55°) and bulk thermal conductivities [1.4 to 30 W/(m·K)] for mechanistic validation, while ensuring broad commercial availability, low cost, and excellent compatibility with water‐based printing systems.

The preparation process involves three steps: (1) Deionized water (CAS#7732‐18‐5, Aladdin) of a required volume (*V*_water_) is added in a beaker and then placed in a cleanroom for 24 h to ensure sufficient air dissolution in the water sample; (2) Nanoparticles of a required mass (*m*_p_) are weighed and dispersed in the water sample and the nanoparticle concentration is *α* = *m*_p_/(*ρ*_p_ *V*_water_) where *ρ*_p_ is the density of the nanoparticles; (3) The nanoparticle‐water (NPW) suspension is subjected to magnetic stirring (500 rpm, JOANLAB‐MSC) for 5 h, followed by an ultrasonic treatment (20 kHz, 600 W, MISONIX‐ Sonicator 3000) for 60 min to break up any potential agglomerates and ensure uniformity. All the above preparation procedures are completed at a 20 ± 1°C room temperature and a 50% ± 5% relative humidity. This standardized protocol is designed to promote air equilibration under controlled ambient conditions, thereby establishing a consistent initial gas‐saturation baseline and improving batch‐to‐batch reproducibility. Moreover, because the solidification time of droplets is usually in the second timescale, which is much shorter than the minutes to hours of aggregation and sedimentation time of nanoparticles in NPW droplets [45], nanoparticles can remain dispersed during the droplet solidification, and no obvious particle agglomeration or sedimentation phenomena are observed during the solidification of NPW droplets.

### 3.2 Preparations of Hele‐Shaw Cells and 2D Droplets

The Hele‐Shaw cell (Figure S26) is composed of two acrylic sidewalls (50 × 40 × 5 mm^3^) and two copper spacers with a 40 mm height and a 0.3 mm thickness. A 2 µL nanoparticle‐water suspension is injected into the gap of the Hele‐Shaw cell using a microliter syringe to form a 2D NPW droplet with an apparent water contact angle of 140°±3°. The Hele‐Shaw cell is then placed on a copper plate and allowed to stand for 5 min to ensure sufficient contact between the droplet and the cold plate. To eliminate potential contamination, all the acrylic components and the copper plate are thoroughly cleaned with absolute ethyl alcohol (CAS#64‐17‐5, 99.5% purity, Aladdin) and deionized water for 5 min each. Subsequently, the acrylic parts and copper plate are dried in a vacuum oven at 50°C and 80°C for 1 h each. A fresh Hele‐Shaw cell is used for each experiment to prevent contamination and residue accumulation.

### 3.3 Experimental Setup for Droplet Solidification

The experimental setup (Figure S26) for droplet solidification includes a temperature control module, an image recording module, and a temperature acquisition module. The temperature control module is used to provide a cold surface for droplet solidification, and it consists of a copper plate with a contact angle of 73°±4° (40 × 40 × 2 mm^3^), a semiconductor cooler, a heat exchanger, a PID controller, a DC power supply, and a constant temperature water bath. The image recording module consists of a high‐speed camera (FASTCAM WX100, with a lens of LEICA Z16), a cold light, and a computer. The temperature acquisition module includes thermocouples (Omega Model T‐TT‐36, accuracy ± 0.5°C) arranged on the upper surface of the cold plate and in the air near the experimental platform to record the cold plate temperature (*T*_c_) and air temperature (*T*_air_) in real time, thermocouples (Kaipusen T‐K‐44‐SLE, accuracy ± 0.1°C) inserted into the droplets to record the droplet nucleation temperature (*T*_n_), a data acquisition instrument (Agilent 34970A), and a computer. To prevent condensation and frosting, the Hele‐Shaw cell is enclosed within a protective chamber, and a low flow of high‐purity nitrogen (CAS#7727‐37‐9, 99.9% purity, Beijing Haipu Beifen Qiti Ind. Ltd. Co.) is fed during experiments. All the droplet solidification experiments are completed at a 20±1°C room temperature and a 50%±5% relative humidity.

### 3.4 Experimental Setup for Mechanics Testing of Solidified Droplets

The experimental setup for mechanical strength testing of solidified droplets (Figure S27) comprises a measurement module and a drive module. This setup performs in‐situ mechanical strength testing on solidified droplets. The measurement module mainly consists of a pressure sensor (DAYSENSOR DYLY‐103, accuracy 0.03%), a data acquisition instrument (DAYSENSOR DY510), and a computer. The drive module is composed of a stepper motor (8 Hz, 0.04 rpm, ZZMOTOR 57HS56‐3004A) and a sliding rail (OnlyPower GX80, accuracy 0.03 mm). The loading velocity of the push rod is 0.16 mm/s, and the strain rate is 0.11 s^−1^. To prevent the pressure sensor operating at room temperature from melting the solidified droplets during measurement, a thermal protective layer is wrapped around the pressure sensor. All the mechanical strength tests of 3D solidified droplets are completed at a 20 ± 1°C room temperature and a 50% ± 5% relative humidity with the cold plate temperature maintained at −20±0.2°C.

### 3.5 Data Processing

During the droplet solidification experiments, the high‐speed camera is set to record images at 1000 frames per second (FPS) at the nucleation and recalescence stage and 50 FPS at the freezing stage with a resolution of 3.98 µm per pixel. Experimental data (e.g., dendrite height, freezing front height, bubble width, bubble length, and bubble position) are quantitatively extracted from images using ImageJ software. To ensure statistical reliability, *N* = 30 independent replicates are performed for the stochastic ice nucleation process, while at least *N* = 5 replicates are conducted for macroscopic freezing and mechanical characterizations (*v*_d_, *v*_f_, *p*, and *σ*_max_), with all error bars throughout the manuscript representing the standard deviation (SD).

## Author Contributions

R.G. and X.Z. contributed equally to this work. R.G., X.Z., M.S., and R.Y. conceived the idea for the work. R.G. and K.S. performed the experiments. X.Z., M.S., R.Y., and J.S. guided the experiments. R.G., X.Z., and K.S. developed the theoretical models, performed the simulations, and analyzed the data. R.G. and X.Z. wrote the manuscript. M.S., K.S., L.Z., and S.Y. edited the manuscript. M.S., Y.G., R.W., and C.C. supervised the work. All authors have approved the final version of the manuscript.

## Conflicts of Interest

The authors declare no conflict of interest.

## Supporting information




**Supporting File 1**: advs76909‐sup‐0001‐SuppMat.docx.

**Supporting File 2**: advs76909‐sup‐0002‐VideoS1.mp4.

**Supporting File 3**: advs76909‐sup‐0003‐VideoS2.mp4.

**Supporting File 4**: advs76909‐sup‐0004‐VideoS3.mp4.

**Supporting File 5**: advs76909‐sup‐0005‐VideoS4.mp4.

**Supporting File 6**: advs76909‐sup‐0006‐VideoS5.mp4.

**Supporting File 7**: advs76909‐sup‐0007‐VideoS6.mp4.

## Data Availability

The data generated in this study are included in the manuscript and supporting information and will be made available from the lead contact upon reasonable request.

## References

[1] S. Florczak , G. Größbacher , D. Ribezzi , et al., “Adaptive and Context‐aware Volumetric Printing,” Nature 645, no. 8079 (2025): 108–114, 10.1038/s41586-025-09436-7. PMC12408377 40903601

[2] J. W. Kim , M. J. Allen , E. A. Recker , et al., “Hybrid Epoxy–acrylate Resins for Wavelength‐Selective Multimaterial 3D Printing,” Nature Materials 24, no. 7 (2025): 1116–1125, 10.1038/s41563-025-02249-z. 40588542

[3] S. Torino , M. Dhurandhar , A. Stroobants , R. Claessens , and R. G. Efremov , “Time‐resolved Cryo‐EM Using a Combination of Droplet Microfluidics With on‐demand Jetting,” Nature Methods 20, no. 9 (2023): 1400–1408, 10.1038/s41592-023-01967-z. 37592181

[4] O. Dadras‐Toussi , B. Hariprasad , and M. R. Abidian , “Multimaterial 3D Printing of Soft and Stretchable Electronics,” Advanced Science 13, no. 8 (2025): 13208, 10.1002/advs.202513208. PMC12884721 41271579

[5] W. Y. Choi , W. Kim , J. R. Choi , et al., “A Hyperelastic Torque‐reversal Mechanism for Soft Joints With Compression‐responsive Transient Bistability,” Science Robotics 10, no. 98 (2025): ado7696, 10.1126/scirobotics.ado7696. 39879278

[6] P. Ferraro , S. Coppola , S. Grilli , M. Paturzo , and V. Vespini , “Dispensing Nano–pico Droplets and Liquid Patterning by PyroElectrodynamic Shooting,” Nature Nanotechnology 5, no. 6 (2010): 429–435, 10.1038/nnano.2010.82. 20453855

[7] S. Pinilla , J. Coelho , K. Li , J. Liu , and V. Nicolosi , “Two‐dimensional Material Inks,” Nature Reviews Materials 7, no. 9 (2022): 717–735, 10.1038/s41578-022-00448-7.

[8] M. J. Song , R. M. Gao , C. B. Dang , K. K. Shao , and L. Zhang , “Solidification Characteristics of Two‐dimensional Water Droplets,” Droplet 4, no. 4 (2025): 70031, 10.1002/dro2.70031.

[9] T. J. K. Buchner , S. Rogler , S. Weirich , et al., “Vision‐controlled Jetting for Composite Systems and Robots,” Nature 623, no. 7987 (2023): 522–530, 10.1038/s41586-023-06684-3. PMC10651485 37968527

[10] C. Suvanjumrat , K. Chansoda , M. Promtong , P. Wiroonpochit , T. Kaewprakob , and W. Chookaew , “Optimization of Drop‐on‐demand 3D Printing of Natural Latex Ink for the Fabrication of Customized Medical Splints,” Cleaner Engineering and Technology 29 (2025): 101112, 10.1016/j.clet.2025.101112.

[11] S. Papazoglou , C. Petridis , E. Kymakis , et al., “In‐situ Sequential Laser Transfer and Laser Reduction of Graphene Oxide Films,” Applied Physics Letters 112, no. 18 (2018): 183301, 10.1063/1.5021862.

[12] S. Sundaram , M. Skouras , D. S. Kim , L. Van Den Heuvel , and W. Matusik , “Topology Optimization and 3D Printing of Multimaterial Magnetic Actuators and Displays,” Science Advances 5, no. 7 (2019): aaw1160, 10.1126/sciadv.aaw1160. PMC6625816 31309144

[13] X. Zhai , Y. Ma , C. Hou , et al., “3D‐Printed High Strength Bioactive Supramolecular Polymer/Clay Nanocomposite Hydrogel Scaffold for Bone Regeneration,” ACS Biomaterials Science & Engineering 3, no. 6 (2017): 1109–1118, 10.1021/acsbiomaterials.7b00224. 33429585

[14] W. C. Liu , V. H. Y. Chou , R. P. Behera , and H. L. Ferrand , “Magnetically Assisted Drop‐on‐demand 3D Printing of Microstructured Multimaterial Composites,” Nature Communications 13, no. 1 (2022): 5015, 10.1038/s41467-022-32792-1. PMC9418172 36028505

[15] M. W. Peng , Z. Wen , L. J. Xie , et al., “3D Printing of Ultralight Biomimetic Hierarchical Graphene Materials With Exceptional Stiffness and Resilience,” Advanced Materials 31, no. 35 (2019): 1902930, 10.1002/adma.201902930. 31267581

[16] N. C. Brown , D. C. Ames , and J. Mueller , “Multimaterial Extrusion 3D Printing Printheads,” Nature Reviews Materials 10, no. 11 (2025): 807–825, 10.1038/s41578-025-00809-y.

[17] D. Kam , O. Rulf , A. Reisinger , R. Lieberman , and S. Magdassi , “3D Printing by Stereolithography Using Thermal Initiators,” Nature Communications 15, no. 1 (2024): 2285, 10.1038/s41467-024-46532-0. PMC10937977 38480705

[18] T. Yang , Y. L. Zhao , Z. B. Jiao , et al., “Multicomponent Intermetallic Nanoparticles and Superb Mechanical Behaviors of Complex Alloys,” Science 362, no. 6417 (2018): 933–937, 10.1126/science.aas8815. 30467166

[19] K. K. Shao , X. Zhang , M. J. Song , et al., “Manipulating Trapped Air Bubbles in Ice for Message Storage in Cold Regions,” Cell Reports Physical Science 6, no. 6 (2025): 102622, 10.1016/j.xcrp.2025.102622.

[20] L. Wu , “The Past and Present of Frozen Bubbles,” Nature Physics 21, no. 4 (2025): 515, 10.1038/s41567-025-02892-y.

[21] C. Y. Kim , D. W. Cho , S. Kim , S. W. Song , K. M. Seo , and Y. T. Cho , “High‐Throughput Metal 3D Printing Pen Enabled by a Continuous Molten Droplet Transfer,” Advanced Science 10, no. 6 (2022): 2205085, 10.1002/advs.202205085. PMC9951324 36526589

[22] J. H. Martin , B. D. Yahata , J. M. Hundley , J. A. Mayer , T. A. Schaedler , and T. M. Pollock , “3D printing of High‐strength Aluminium Alloys,” Nature 549, no. 7672 (2017): 365–369, 10.1038/nature23894. 28933439

[23] P. Brahana , M. Zhang , E. Nakouzi , and B. Bharti , “Weathering Influences the Ice Nucleation Activity of Microplastics,” Nature Communications 15, no. 1 (2024): 9579, 10.1038/s41467-024-53987-8. PMC11542094 39505887

[24] A. G. Marín , O. R. Enríquez , P. Brunet , P. Colinet , and J. H. Snoeijer , “Universality of Tip Singularity Formation in Freezing Water Drops,” Physical Review Letter 113 (2014): 054301, 10.1103/PhysRevLett.113.054301. 25126922

[25] R. Huang , X. Zhang , L. Zhang , et al., “Icing Characteristics of Micropillar‐encapsulated Sessile Water Droplets on Cold Surfaces,” International Journal of Heat and Mass Transfer, 261 (2026): 128527, 10.1016/j.ijheatmasstransfer.2026.128527.

[26] L. Sri SV , A. Karthick , and C. Dinesh , “Evaluation of Mechanical Properties of 3D Printed PETG and Polyamide (6) Polymers,” Chemical Physics Impact 8 (2024): 100491, 10.1016/j.chphi.2024.100491.

[27] S. Nathaphan and W. Trutassanawin , “Effects of Process Parameters on Compressive Property of FDM With ABS,” Rapid Prototyping Journal 27, no. 5 (2021): 905–917, 10.1108/RPJ-12-2019-0309.

[28] V. Pruthi , V. Hirschberg , and P. Theato , “3D Printable Polyelectrolyte Complex‐Integrated Interpenetrating Network Hydrogels With Customizable Mechanical Strength and pH‐Responsiveness,” Advanced Materials Technologies 9, no. 13 (2024): 2400416, 10.1002/admt.202400416.

[29] Y. A. Orozco‐Osorio , A. V. Gaita‐Anturi , C. P. Ossa‐Orozco , et al., “Utilization of Additive Manufacturing Techniques for the Development of a Novel Scaffolds With Magnetic Properties for Potential Application in Enhanced Bone Regeneration,” Small 20, no. 44 (2024): 2402419, 10.1002/smll.202402419. 39004887

[30] H. Liu , S. Laflamme , A. Cardinali , et al., “Enhancing 3D‐printed Cementitious Composites With Recycled Carbon Fibers From Wind Turbine Blades,” Construction and Building Materials 472 (2025): 140650, 10.1016/j.conbuildmat.2025.140650.

[31] C. Cui , Z. Y. Zhuang , H. L. Gao , J. Pang , X. F. Pan , and S. H. Yu , “3D printing of Ultrahigh Filler Content Composites Enabled by Granular Hydrogels,” Advanced Materials 37, no. 30 (2025): 2500782, 10.1002/adma.202500782. 40347044

[32] K. Christensen , B. Davis , Y. Jin , and Y. Huang , “Effects of Printing‐induced Interfaces on Localized Strain Within 3D Printed Hydrogel Structures,” Materials Science and Engineering: C 89 (2019): 65–74, 10.1016/j.msec.2018.03.014. 29752120

[33] Z. S. Zhang and X. Y. Liu , “Control of Ice Nucleation: Freezing and Antifreeze Strategies,” Chemical Society Reviews 47, no. 18 (2018): 7116–7139, 10.1039/C8CS00626A. 30137078

[34] I. Hwang , Z. Guan , C. Cao , W. L. Tang , C. O. Chui , and X. C. Li , “Nanoparticles Suppress Fluid Instabilities in the Thermal Drawing of Ultralong Nanowires,” Nature Communications 11, no. 1 (2020): 5932, 10.1038/s41467-020-19796-5. PMC7683681 33230110

[35] G. Y. Bai , D. Gao , Z. Liu , X. Zhou , and J. J. Wang , “Probing the Critical Nucleus Size for Ice Formation With Graphene Oxide Nanosheets,” Nature 576, no. 7787 (2019): 437–441, 10.1038/s41586-019-1827-6. 31853083

[36] S. J. Cox , S. M. Kathmann , B. Slater , and A. Michaelides , “Molecular Simulations of Heterogeneous Ice Nucleation. I. Controlling Ice Nucleation Through Surface Hydrophilicity,” The Journal of Chemical Physics 142, no. 18 (2015): 184704, 10.1063/1.4919714. 25978902

[37] T. F. Whale , M. Rosillo‐Lopez , B. J. Murray , and C. G. Salzmann , “Ice Nucleation Properties of Oxidized Carbon Nanomaterials,” The Journal of Physical Chemistry Letters 6, no. 15 (2015): 3012–3016, 10.1021/acs.jpclett.5b01096. PMC4550106 26267196

[38] M. Fitzner , P. Pedevilla , and A. Michaelides , “Predicting Heterogeneous Ice Nucleation With a Data‐driven Approach,” Nature Communications 11, no. 1 (2020): 4777, 10.1038/s41467-020-18605-3. PMC7509812 32963232

[39] G. Karunakaran , R. Suriyaprabha , V. Rajendran , and N. Kannan , “Effect of Contact Angle, Zeta Potential and Particles Size on the in Vitro Studies of Al_2_O_3_ and SiO_2_ Nanoparticles,” IET Nanobiotechnology 9, no. 1 (2015): 27–34, 10.1049/iet-nbt.2013.0067. 25650323

[40] J. G. Vlahakis and A. J. Barduhn , “Growth rate of an ice crystal in flowing water and salt solutions,” AIChE Journal 20, no. 3 (1974): 581–591, 10.1002/aic.690200320.

[41] S. W. Churchill and H. H. S. Chu , “Correlating Equations for Laminar and Turbulent Free Convection From a Vertical Plate,” International Journal of Heat and Mass Transfer 18, no. 11 (1975): 1323–1329, 10.1016/0017-9310(75)90243-4.

[42] K. K. Shao , M. J. Song , B. H. Cai , L. Zhang , L. Pekar , and X. Zhang , “Effects of Environmental Temperature and Trapped Air Bubbles on the Mechanical Properties of Ice Cubes,” ACS Applied Materials & Interfaces 16, no. 46 (2024): 63482–63494, 10.1021/acsami.4c12227. 39509341

[43] S. Saher , S. Johnston , R. Esther‐Kelvin , J. M. Pringle , D. R. MacFarlane , and K. Matuszek , “Trimodal Thermal Energy Storage Material for Renewable Energy Applications,” Nature 636, no. 8043 (2024): 622–626, 10.1038/s41586-024-08214-1. 39695206

[44] X. Lyu , W. Lei , G. Gardi , et al., “Optofluidic Three‐dimensional Microfabrication and Nanofabrication,” Nature 650, no. 8102 (2026): 613–620, 10.1038/s41586-025-10033-x. PMC12916288 41606333

[45] Y. J. Yang , D. S. Corti , and E. I. Franses , “Use of Close‐packed Vesicular Dispersions to Stabilize Colloidal Particle Dispersions Against Sedimentation,” Langmuir 31, no. 32 (2015): 8802–8808, 10.1021/acs.langmuir.5b02133. 26203879

