# Supplementary material for: Nanoparticle‐Mediated Bubble Suppression During Droplet Solidification for Mechanical Reinforcement
Source: Adv Sci (Weinh). 2026 Aug 5;13(59):e76909. doi: 10.1002/advs.76909 (PMC13440213; doi:10.1002/advs.76909)
Supplement: Supplementary file 1 [file ADVS-13-e76909-s007.docx]

**Supporting Information**

Nanoparticle-Mediated Bubble Suppression during Droplet Solidification for Mechanical Reinforcement

Runmiao Gao^1,2,†^, Xuan Zhang^1,†^, Mengjie Song^1,*^, Ronggui Yang^3^, Keke Shao^1^, Jun Shen^1^, Long Zhang^1^, Shuhuai Yao^4^, Yubing Guo^5^, Ruzhu Wang^6^, Christopher Yu Hang Chao^7,8^

^1^ Department of Energy and Power Engineering, School of Mechanical Engineering,

Beijing Institute of Technology, Beijing 100081, China

^2^ Department of Mechanical Engineering, The University of Tokyo, Tokyo 113-8656, Japan

^3^ School of Mechanics and Engineering Science, Peking University, Beijing 100871, China

^4^ Department of Mechanical and Aerospace Engineering, The Hong Kong University of Science and Technology, Kowloon, Hong Kong 999077, China

^5^ School of Medical Technology, Beijing Institute of Technology, Beijing 100081, China

^6^ Institute of Refrigeration and Cryogenics, MOE Engineering Research Center of Solar Power & Refrigeration, Shanghai Jiao Tong University, Shanghai 200240, China

^7^ Department of Building Environment and Energy Engineering, The Hong Kong Polytechnic University, Hong Kong 999077, China

^8^ Department of Mechanical Engineering, The Hong Kong Polytechnic University, Hong Kong 999077, China

†Runmiao Gao and Xuan Zhang contributed equally to this work.

*Corresponding author: Mengjie Song

Tel: +86-188 1172 6830, E-mail: mengjie.song@bit.edu.cn

Figure S1. Real-time visual observation of droplet solidification features.

**(A)** Schematic diagram of the Hele-Shaw cell for the solidification experiments of two-dimensional (2D) deionized water (DIW) and nanoparticle-suspension water (NPW) droplets. The gap width of the Hele-Shaw cell is 0.3 mm with *V*_droplet_ and *T*_c_ being the droplet volume and cold plate temperature.

**(B-C)** Experimental images of the solidification process of 3D and 2D DIW droplets. A 3D DIW droplet hampers a clear observation of internal features during solidification, such as dendrites, the freezing front, and trapped air bubbles in the ice. In contrast, these solidification features are distinctly observable in a 2D DIW droplet. The volumes of the 3D and 2D DIW droplets are 10 μL and 2 μL. The scale bar is 1 mm.

Figure S2. Time evolution of internal droplet temperature during solidification.

**(A)** Schematic diagram of the time evolution of internal droplet temperature during solidification. *T*_0_, *T*_f_, and *T*_n_ are the initial, freezing, and nucleation temperatures, respectively. Based on the temperature change curve, the droplet solidification process can be divided into four stages, i.e., supercooling stage, nucleation and recalescence stage, freezing stage, and cooling stage.

**(B-C)** Time evolutions of internal temperatures during the solidification processes of 2D DIW and NPW droplets and 3D ones. NPW droplets contain TiO_2_ nanoparticles with a 20 nm diameter and a 0.01 vol% concentration. The volumes of the 2D and 3D droplets are 2 μL and 10 μL. The distances from the thermocouple to the bottom surface and the central axis of the droplet are 0.18 mm and 0.3 mm. The scale bar is 1 mm. The lowest temperature at the end of the supercooling stage indicates the nucleation temperature. The nucleation temperatures of 3D and 2D NPW droplets are elevated from -12.72 °C and -18.95 °C to -9.77 °C and -16.39 °C, by 23.19% and 13.51%.

Figure S3. Distribution characteristics of trapped air bubbles in solidified DIW and NPW droplets.

**(A)** Schematic illustration of the freezing stage of an NPW droplet. The advancing freezing front is accompanied by the rejection of nanoparticles and the formation of trapped air bubbles in ice. *H*_f_ and *H*_b_ are the heights of the freezing front and trapped air bubble with *L*_b_ and *W*_b_ being the bubble length and width.

**(B)** Experimental images of solidification processes of DIW and TiO_2_-NPW droplets at *T*_c_ = -20 °C. At the freezing stage, the freezing front is advancing with the concomitant formation of trapped air bubbles. The addition of TiO_2_ nanoparticles prolongs the solidification duration of the droplets from 38 s to 65 s, increasing by 71.05%, and reduces the number of trapped air bubbles as the size increases. The scale bar is 1 mm.

**(C)** Variation of the width and aspect ratio (*AR* = *L*_b_/*W*_b_) of trapped air bubbles with bubble height in solidified DIW and TiO_2_-NPW droplets. Both the bubble width and aspect ratio generally increase with the increase of bubble height. At the same height, the TiO_2_-NPW droplet always generates a larger bubble width and aspect ratio. The addition of nanoparticles enlarges the slope of the bubble width curve from 10.25 s to 22.17 μm/mm, increasing by 116.29%, with the average bubble width and *AR* increasing from 30.43 μm to 52.15 μm and from 1.80 to 2.01.

**(D)** Variation of the number (*N*_b_) and volume (*V*_b_) of trapped air bubbles with bubble height in solidified DIW and TiO_2_-NPW droplets. With the increase of bubble height, the bubble number basically decreases first and then increases while the bubble volume generally increases. The addition of nanoparticles reduces the average bubble number from 13 to 6, decreasing by 53.85%, and increases the average bubble volume from 16,663.68 μm^3^ to 32,017.44 μm^3^, increasing by 92.14%.

Figure S4. Nanoparticle effect on nucleation temperature of DIW and NPW droplets.

**(A)** Nucleation temperature distributions at different nanoparticle concentrations, diameters, and types.

**(B)** Probability (*p*_n_) distribution histogram of nucleation temperature under various nanoparticle concentrations, diameters, and types. As the nanoparticle concentration increases and the nanoparticle diameter decreases, the peak of nucleation probability shifts to the high-temperature side. The nucleation temperature at the peak of nucleation probability for Al_2_O_3_-NPW droplets is the highest compared to other nanoparticles.

**(C)** Proportion (*P*_n_) of nucleated droplets at different nanoparticle concentrations, diameters, and types.

Figure S5. Schemes of the hydrogen bonding networks at the interfacial regions and the corresponding intrinsic contact angle (*θ*_int_) of (A) SiO_2_ and (B) Al_2_O_3_.

From a microscopic perspective, the specific chemical nature of the nanoparticle establishes the local atomic template and charge distribution for molecular ordering ^[1]^. Different oxide types possess distinct surface atomic configurations, such as varying densities of surface hydroxyl groups (-OH) and localized coordination sites ^[2]^. On a molecular scale, the surface chemistry of highly hydrophilic oxides like Al_2_O_3_ inherently allows it to actively foster a significantly tighter, denser, and highly organized interfacial hydrogen-bond network with the surrounding supercooled water molecules compared to oxides like SiO_2_ ^[3]^. This robust hydrogen-bond network stabilizes nascent, ice-like structural clusters near the phase boundary, acting as a microscopic thermodynamic catalyst for crystal embryo formation ^[3,4]^.

From a macroscopic perspective, these microscopic chemical variations manifest directly as bulk thermodynamic behaviors that can be characterized via boundary energy balances and wetting metrics ^[5]^. Here, the intrinsic contact angle (*θ*_int_) serves as the primary macroscopic indicator of the underlying surface chemistry ^[6]^, exhibiting a distinct experimental gradient ranging from 35° for Al_2_O_3_ to 55° for SiO_2_ ^[7]^. By employing Young’s equation (*γ*_pw_ = *γ*_pg_ – *γ*_gw_cos*θ*_int_), this macroscopic parameter determines the nanoparticle-water interfacial tension (*γ*_pw_) ^[8]^. Consequently, the distinct surface chemistry of each nanoparticle type imposes a deterministic shift on the bulk interfacial tension balance at the multiphase boundary, representing the macroscopic thermodynamic manifestation of the constituent material constraints ^[7,8]^.

From a micro-macro coupling perspective, the regulating effect of nanoparticle chemistry operates as an interconnected, cross-scale chain where microscopic hydrogen bonding serves as the causal molecular driver ^[6,9]^, and macroscopic surface parameters serve as the thermodynamic consequence ^[5]^. Specifically, the formation of a tighter microscopic hydrogen-bond network (micro-scale cause) alters the local molecular arrangements, which macroscopically manifests as a reduced intrinsic contact angle and modulated interfacial tension (*γ*_pw_), as shown in **Figure S5**. As solid nanoparticles substitute water molecules at the phase boundary, this tension shift directly lowers the effective ice-water interfacial energy (*γ*_iw_). According to Classical Nucleation Theory, this reduction in *γ*_iw_ exponentially minimizes the thermodynamic Gibbs free energy barrier (Δ*G*^*^) ^[10]^. By minimizing Δ*G*^*^, the nascent ice nucleus is stabilized at a much lower degree of supercooling, which ultimately elevates the macroscopic nucleation temperature (*T*_n_) ^[11]^. Therefore, nanoparticle chemistry regulates solidification kinetics through a mechanism: microscopic hydrogen bonding drives the macroscopic surface tension states, which in turn dictate the ultimate phase-change thermodynamics.

Figure S6. Variations of nucleation temperature and effective ice-water interfacial energy with intrinsic contact angle of nanoparticles.

The intrinsic contact angles (*θ*_int_) of Al_2_O_3_, TiO_2_, Fe_2_O_3_, and SiO_2_ nanoparticles are 35°, 40°, 50°, and 55°, respectively. As the intrinsic contact angle of nanoparticles decreases, the nucleation temperature is elevated and the ice-water interfacial energy (*γ*_iw_) is reduced. Al_2_O_3_-NPW and SiO_2_-NPW droplets yield the highest and lowest average nucleation temperatures of -21.47 °C and -23.21 °C.

Figure S7. Variations of nanoparticle influencing factor with nanoparticle concentration and diameter for (A) Al_2_O_3_, (B) TiO_2_, (C) Fe_2_O_3_, and (D) SiO_2_.

Figure S8. Dendrite structures in DIW and NPW droplets at the end of the nucleation and recalescence stage at *T*_c_ = -20 °C.

**(A)** Effect of nanoparticle concentration for TiO_2_ nanoparticles with a 50 nm diameter. The duration of the nucleation and recalescence stage is reduced to 72 ms at a 0.1 vol% concentration, a significant decrease of 40% compared to the duration of 120 ms for a DIW droplet. As the nanoparticle concentration increases, the dendrite height decreases. The scale bar is 1 mm.

**(B)** Effect of nanoparticle diameter for TiO_2_ nanoparticles with a 0.01 vol% concentration. As the nanoparticle diameter increases from 20 nm to 100 nm, the duration rises from 76 ms to 104 ms, by 36.84%. As the nanoparticle diameter increases, the dendrite height rises.

**(C)** Effect of nanoparticle type with a 50 nm diameter and a 0.01 vol% concentration. Al_2_O_3_-NPW droplets yield the shortest duration of the nucleation and recalescence stage and the lowest dendrite height.

Figure S9. Time evolution of average dendrite height in DIW and NPW droplets during nucleation and recalescence stage.

**(A)** Effect of nanoparticle concentration for TiO_2_ nanoparticles with a 50 nm diameter. The average dendrite height is reduced to 1.05 mm at 0.1 vol% concentration, a reduction of 49.27% compared to the dendrite height of 2.07 mm for the DIW droplet.

**(B)** Effect of nanoparticle diameter for TiO_2_ nanoparticles with a 0.01 vol% concentration. As the nanoparticle diameter decreases from 100 nm to 20 nm, the average dendrite height reduces from 1.72 mm to 1.13 mm, by 34.3%.

**(C)** Effect of nanoparticle type with a 50 nm diameter and a 0.01 vol% concentration. For the Al_2_O_3_-NPW droplet, the average dendrite height is reduced to 1.17 mm, a significant reduction of 28.16% compared to the dendrite height of 1.63 mm for SiO_2_-NPW droplet.

Figure S10. Dendrite gaps under various nanoparticle concentrations, diameters, and types at the end of the nucleation and recalescence stage.

**(A)** Dendrite gap (*G*) versus dendrite number (*n*) under various nanoparticle concentrations, diameters, and types. The measurement position of dendrite gap is 0.8 mm above the cold plate.

**(B)** Variation of dendrite gap with nanoparticle concentrations, diameters, and types. The dendrite gap exhibits a negative and positive correlation with the nanoparticle concentration and nanoparticle diameter, while the TiO_2_-NPW droplets yield the largest dendrite gap.

Figure S11. Experimental images of freezing process of (A) DIW, (B) TiO_2_-α3-D50, (C) TiO_2_-α2-D50, and (D) TiO_2_-α1-D50 NPW droplets under different nanoparticle concentrations at the freezing stage.

The scale bar is 1 mm. The addition of nanoparticles prolongs the duration of the freezing stage. At the 0.1 vol% concentration, there are larger but fewer trapped bubbles in the solidified droplet compared to the DIW droplet.

Figure S12. Experimental images of freezing process of (A) TiO_2_-α2-D20, (B) TiO_2_-α2-D50, and (C) TiO_2_-α2-D100 NPW droplets under different nanoparticle diameters at the freezing stage.

The scale bar is 1 mm. As the nanoparticle diameter decreases, the duration of the freezing stage increases, the size of trapped air bubbles increases, and the number of trapped air bubbles decreases.

Figure S13. Experimental images of freezing process of (A) Al_2_O_3_-α2-D50, (B) TiO_2_-α2-D50, (C) Fe_2_O_3_-α2-D50, and (D) SiO_2_-α2-D50 NPW droplets under different nanoparticle types in the freezing stage.

The scale bar is 1 mm. The Al_2_O_3_-NPW droplets yield the longest duration of freezing stage and the largest trapped bubble.

Figure S14. Droplet shape and deformation during freezing stage under various nanoparticle (A) concentrations, (B) diameters, and (C) types.

The dynamic morphological profiles of the droplets during solidification on a -20 °C cold plate are illustrated. As illustrated in **Figure S14A**, increasing the nanoparticle concentration (from DIW to TiO_2_-α3-D50 and TiO_2_-α2-D50) significantly alters the macroscopic deformation, inducing enhanced lateral outward expansion and a progressive suppression of the frozen pointy tip. Specifically, DIW terminates with a sharp pointy tip singularity at 38 s due to the instantaneous axial eruption of expansion stress driven by the intrinsic ice-to-water density mismatch; however, the tip becomes noticeably blunted at a lower concentration (TiO_2_-α3-D50, 41 s) and transforms into a smooth, flat plateau at a higher concentration (TiO_2_-α2-D50, 59 s). Furthermore, **Figure S14B** demonstrates that the nanoparticle diameter plays a critical role in modulating the freezing kinetics and structural relaxation, where smaller nanoparticles (TiO_2_-α2-D20) significantly prolong the total freezing time to 65 s while maintaining a highly flattened top, whereas larger diameters (TiO_2_-α2-D100) accelerate the solidification process to 45 s. Crucially, as evidenced in **Figure S14C**, this pointy-tip suppression behavior is demonstrated to be a universal phenomenon across various nanoparticle types (Al_2_O_3_-α2-D50, Fe_2_O_3_-α2-D50, and SiO_2_-α2-D50), all of which consistently eliminate the singular tip to form stable, flattened profiles, with their total freezing times (ranging from 51 s to 67 s) governed by the distinct material-dependent thermal conductivities and ice-nucleation pinning effects of the respective nanofluid systems.

Figure S15. Time evolution of the freezing front height of DIW and NPW droplets during the freezing stage.

**(A)** Effect of nanoparticle concentration for TiO_2_ nanoparticles with a 50 nm diameter. The duration of the freezing stage is extended to 57 s at 0.1 vol% concentration, an increase of 50% compared to the duration of 38 s for the DIW droplet.

**(B)** Effect of nanoparticle diameter for TiO_2_ nanoparticles with a 0.01 vol% concentration. As the nanoparticle diameter decreases from 100 nm to 20 nm, the freezing-stage duration increases from 45 s to 65 s, by 44.44%.

**(C)** Effect of nanoparticle type with a 50 nm diameter and a 0.01 vol% concentration. The Al_2_O_3_-NPW droplets yield the longest duration of the freezing stage.

Figure S16. Changes of freezing rate and bubble width with freezing front height under various nanoparticle (A) concentrations, (B) diameters, and (C) types.

The freezing rate gradually decreases with the advancement of the freezing front, which in turn leads to an increase in the bubble width. The addition of nanoparticles decreases the freezing rate and increases the bubble width.

Figure S17. Changes of bubble size with bubble height under various nanoparticle concentrations, diameters, and types.

**(A)** Bubble width. The bubble width generally increases with the increase of the bubble height.

**(B)** Bubble length. The bubble length generally increases with the increase of the bubble height.

**(C)** Bubble equivalent diameter. The bubble equivalent diameter is calculated as *D*_b_ = (6*V*_b_/π)^1/3^. The bubble equivalent diameter generally increases with the increase of the bubble height.

Figure S18. Transient growth dynamics of bubble size over time at a height of 2.7 mm. (A) Absolute bubble width and length, and (B) Normalized bubble width and normalized growth time.

Regarding the transient growth dynamics captured at a height of 2.7 mm, the absolute bubble width (*W*_b_) and length (*L*_b_) exhibit highly coupled expansion histories governed by localized gas diffusion. Qualitatively, as solidification proceeds, gas molecules rejected by the ice accumulate continuously ahead of the advancing front, driving the rapid initial expansion of the bubble envelope. Quantitatively, the introduction of nanoparticles significantly elongates the absolute life cycle of bubble growth. For DIW droplets, the total transient growth duration is limited to approximately 3.5 s. However, for TiO_2_-α1-D50 droplets, the growth duration broadens substantially to 4.5 s. While the transverse width *W*_b_ demonstrates a parabolic trajectory that peaks around 70-80 μm before terminating due to ice encapsulation, the longitudinal length *L*_b_ shows an accelerating, near-linear expansion that reaches 100-200 μm. Strikingly, when these transient profiles are non-dimensionalized by their respective maximum values ($W_{\text{b}}^{'}=W_{\text{b}}/W_{\text{b,max}}$and $t^{'}=t_{\text{b}}/t_{\text{b,max}}$), the data points across all concentration, diameter, and type gradients collapse cleanly onto a unified evolutionary curve situated just below the 1:1 diagonal line. This data collapse proves that despite changes in absolute time scales, the interfacial gas expansion obeys a highly conserved, self-similar scaling law mediated strictly by the localized mass diffusion boundary layer.

Figure S19. Spatial variations of the trapped air bubble aspect ratio along the bubble height under various nanoparticle (A) concentrations, (B) diameters, and (C) types.

Regarding the spatial geometry of the bubble envelope along the droplet height, the aspect ratio (*L*_b_/*W*_b_) exhibits a strong spatial dependence coupled with freezing front kinetics. Qualitatively, near the cold base (*H*_b_ ≈ 0.2 mm), the bubbles are initiated with an aspect ratio close to 1.4, indicating a more spherical morphology due to the rapid advancement of the freezing front which quickly freezes the trapped bubbles. As the bubble height *H*_b_ increases toward the middle-upper region (*H*_b_ ≈ 2.0-2.5 mm), the aspect ratio exhibits a continuous upward trajectory, peaking between 2.2 and 2.6 before undergoing a subtle saturation at the very top of the droplet. Quantitatively, the addition of nanoparticles, specifically higher concentrations, smaller diameters, or highly hydrophilic types like Al_2_O_3_, shifts the entire aspect ratio curve upward compared to the DIW droplets. This morphology transition occurs because the nanoparticle-enhanced thermal conductivity effectively suppresses the freezing rate, granting the expelled air bubbles significantly more time to undergo axial mass diffusion along the directional temperature gradient before being encapsulated, thereby morphing the trapped bubbles from spheres into elongated ellipsoids.

Figure S20. Experimental images of mechanical compression tests of 3D solidified (A) DIW and (B) NPW droplets.

The scale bar is 1 mm. The first-crack point is reached when cracks appear on the solidified droplet, and the compressive strength is reached when the solidified droplet completely fails.

Figure S21. Stress-strain curves of solidified 3D solidified droplets under various nanoparticle (A) concentrations, (B) diameters, and (C) types.

**(A)** Effect of nanoparticle concentration for TiO_2_ nanoparticles with a 50 nm diameter. The maximum compressive stress of the solidified droplet increases to 6.11 MPa at 0.1 vol% concentration, an increase of 20.51% compared to the maximum compressive stress of 5.07 MPa for the DIW droplet.

**(B)** Effect of nanoparticle diameter for TiO_2_ nanoparticles with a 0.01 vol% concentration. As the nanoparticle diameter decreases from 100 nm to 20 nm, the maximum compressive stress of the solidified droplet increases from 5.77 MPa to 7.07 MPa, by 22.53%.

**(C)** Effect of nanoparticle type with a 50 nm diameter and a 0.01 vol% concentration. The TiO_2_-NPW droplets yield the highest maximum compressive stress.

Figure S22. Equivalent stress distribution contour maps of solidified (A) DIW, (B) SiO_2_-α2-D50, (C) TiO_2_-α2-D50, and (D) TiO_2_-α2-D20 NPW droplets.

The scale bars in full and magnified views are 0.5 mm and 0.2 mm. A constant force of *F* = 1 N is applied to the sample droplet.

Figure S23. Experimental images of mechanical compression tests of multiple formed (A) DIW and (B) NPW droplets stacked horizontally. The scale bar is 1 mm.

Figure S24. Experimental images of mechanical compression tests of multiple formed (A) DIW and (B) NPW droplets stacked vertically. The scale bar is 1 mm.

Figure S25. Experimental images of mechanical compression tests of formed FL and BIT patterns with DIW and NPW droplets.

**(A)** FL pattern with DIW droplets. The scale bar is 5 mm.

**(B)** FL pattern with NPW droplet.

**(C)** BIT pattern with DIW droplets.

**(D)** BIT pattern with NPW droplets.

Figure S26. Schematic diagram of experimental setup for droplet solidification.

**(A)** Experiment setup for 2D droplet solidification in a Hele-Shaw cell.

**(B)** Details of the Hele-Shaw cell and 2D droplet.

**(C)** Time evolution of cold plate temperatures during rapid cooling and slow cooling, as well as the environmental temperature. Slow cooling of the cold plate is employed to analyze the droplet nucleation temperature, with a cooling rate of -6.28 °C/min. Rapid cooling of the cold plate is used to analyze droplet solidification characteristics, at a cooling rate of -1.17 °C/s, until the cold plate temperature is held at -20±0.2 °C.

Figure S27. Schematic diagram of experimental setup for mechanical compression test.

The in situ mechanical strength measurements of solidified 3D droplets are performed. To prevent the pressure sensor operating at room temperature from melting the solidified droplets during measurement, a thermal-protective layer is wrapped around the pressure sensor.

Note S1. Area fraction occupied by nanoparticles

Given that the volume fraction of nanoparticles is *α* and the nanoparticle diameter is *D*, the nanoparticle number per unit volume in a DIW droplet is calculated by

$N_{\text{b,V}}=\frac{6\alpha}{\text{π}D^{3}}$ **(S1)**

For a uniformly distributed three-dimensional nanoparticle group, each particle occupies an average spatial volume of 1/*N*_b,V_. Assuming the centers of nanoparticles form a simple cubic packing in space with an average interparticle distance of *b*, each nanoparticle occupies a volume of *b*^3^. Therefore,

$b=\left(\frac{1}{N_{\mathrm{b},\mathrm{V}}}\right)^{\frac{1}{3}}$ **(S2)**

In a cubic packing, since the interparticle center distance is *b*, there are 1/*b* nanoparticles in each row and 1/*b* nanoparticles in each column per unit area. Therefore, the number of nanoparticles per unit area can be calculated as

$N_{\text{b,S}}=\frac{1}{b^{2}}$ **(S3)**

Combining **Eqs. S2** and **S3** yields

**(S4)**

Substituting **Eq. S1** into **Eq. S4** yields

$N_{\text{b,S}}=\left(\frac{6\alpha}{\text{π}D^{3}}\right)^{\frac{2}{3}}$ **(S5)**

Therefore, the area fraction occupied by nanoparticles per unit area can be estimated by

$f_{\mathrm{p}}=\frac{6^{\frac{2}{3}}\text{π}^{\frac{1}{3}}\alpha^{\frac{2}{3}}}{4}\sim\alpha^{\frac{2}{3}}$ **(S6)**

Note S2. Calculation of ice-water interfacial energy during droplet nucleation

Nucleation rate is the number of ice nuclei formed per unit time, and it is considered to be the main parameter for studying nucleation behavior. Based on the classical nucleation theory, a method to calculate the nucleation rate from the nucleation temperature is proposed by Seeley and Seidle ^[12]^. Firstly, the nucleation temperature is divided into several intervals with lengths of Δ*T_i_*. The middle value *T_i_* of each temperature interval is used as the characteristic temperature. *m_i_* is the number of nucleation times when the nucleation temperature is within the interval [*T_i_* – Δ*T_i_*/2, *T_i_* + Δ*T_i_*/2]. *r* is the cooling rate of the cold plate surface. According to the physical definition of nucleation rate, the nucleation rate in each temperature interval can be calculated as

$J\left(T_{i}\right)=\frac{m_{i}|r|}{\Delta T_{i}\left(\frac{m_{i}}{2}+\sum_{j>i} m_{j}\right)}$ **(S7)**

**S27** demonstrates the variation of nucleation rate with nanoparticle concentrations (*α*), diameters (*D*), and types. The discrete distribution of nucleation rate calculated by **Eq. S7** can be used to fit a continuous nucleation rate curve. For supercooled water droplets on cold surfaces, the heterogeneous nucleation rate given by classical nucleation theory is ^[13]^

$J\left(T\right)=J_{0}\exp\left[\frac{-\Gamma}{T\left(T_{\text{f}}-T\right)^{2}}\right]$ **(S8)**

in which

$\Gamma=\frac{16\text{π}\gamma_{\text{iw}}^{3}T_{\text{f}}^{2}f\left(\theta_{\mathrm{Y}}\right)}{3\lambda_{\text{B}}\Delta H_{\text{V}}^{2}}$ **(S9)**

where *J*_0_ is the factor in front of the exponential phase in the nucleation rate equation, *γ*_iw_ is the effective interfacial energy between ice and NPW, *T*_f_ = 273.15 K is the freezing temperature, *λ*_B_ = 1.38×10^-23^ is the Boltzmann constant, Δ*H*_V_ = 306 MJ/m^3^ is the volumetric enthalpy of ice melting,^[14]^ and *f*(*θ*_Y_) is the Fletcher factor, i.e.,

$f\left(\theta_{\mathrm{Y}}\right)=\frac{\left(2+\cos\theta_{\mathrm{Y}}\right)\left(1-\cos\theta_{\mathrm{Y}}\right)^{2}}{4}$ **(S10)**

where *θ*_Y_ is Young’s contact angle of the basement. *f*(*θ*_Y_) ranges from 0 to 1 and a value of 0.15 is adopted in this study. The logarithmic form of **Eq. S8** is

$\text{l}\mathrm{og}_{10}\left[J_{\text{hete}}\left(T\right)\right]=\log_{10}\left(J_{0}\right)-\frac{\Gamma}{2.303T\left(T_{\text{f}}-T\right)^{2}}$ **(S11)**

**Eq. S11** can be used to fit a continuous equation of the nucleation rate (**Figure S28**). The fitting parameters for nucleation rates at different nanoparticle concentrations, diameters, and types are shown in **Table S1**. From **Eq. S9**, *γ*_iw_ can be calculated by

$\gamma_{\text{iw}}=\left[\frac{3\lambda_{\text{B}}\Gamma\Delta H_{\text{V}}^{2}}{16\text{π}T_{\text{f}}^{2}f\left(\theta_{\mathrm{Y}}\right)}\right]^{\frac{1}{3}}$ **(S12)**

The value of *γ*_iw_ we calculated is shown in **Table S1**, and it is within a normal range ^[15]^. In the NPW droplets, nanoparticles substitute part of water molecules at the ice-water interface, and the effective interfacial energy is reduced from *γ*_iw,0_ to *γ*_iw_ where *γ*_iw,0_ is the interfacial energy between ice and pure water, described by *γ*_iw_ = *γ*_iw,0_ – *γ*_pw_*f*_p_, where *γ*_pw_ is the interfacial energy between nanoparticles and pure water and *f*_p_ is the area fraction occupied by nanoparticles. Then, the ice-water interfacial energy can be described as

$\gamma_{\text{iw}}=\gamma_{\text{iw,0}}-f_{\mathrm{p}}\left(\gamma_{\text{pg}}-\gamma_{\text{gw}}\cos\theta_{\mathrm{int}}\right)$ **(S13)**

where *θ*_int_ is the intrinsic contact angle of nanoparticles ^[16]^, and *γ*_iw,0_ uses the experimental value of 27.34 mJ/m^2^. The theoretically calculated values for each parameter are shown in **Table S2**. The theoretical and experimental values for the ice-water interface energy are compared as shown in **Figure S29**. The relative error between the theoretical and experimental values of the ice-water interface energy is within 15%. This validates the accuracy of the results for the ice-water interface energy obtained from our theoretical calculations.

**Figure S28. Changes of nucleation rates with nucleation temperature under various nanoparticle (A) concentrations and (B) types.**

**Figure S29. Comparison of theoretical and experimental values for ice-water interface energy.**

**Table S1 Fitting parameters of nucleation rate.**

| Case No. | *α* (vol%) | *D* (nm) | Type | log_10_*J*_0_ | Γ (K^3^) | *γ*_iw_ (mJ/m^2^) | *R*^2^ |
| --- | --- | --- | --- | --- | --- | --- | --- |
| 1 | / | / | / | 7.031 | 1,976,183 | 27.34 | 0.97 |
| 2 | 0.001 | 50 | TiO_2_ | 6.982 | 1,905,277 | 27.01 | 0.97 |
| 3 | 0.01 | 50 | TiO_2_ | 6.769 | 1,692,380 | 25.96 | 0.96 |
| 4 | 0.1 | 50 | TiO_2_ | 5.595 | 1,284,913 | 23.69 | 0.85 |
| 5 | 0.01 | 50 | Al_2_O_3_ | 6.328 | 1,558,184 | 25.26 | 0.87 |
| 6 | 0.01 | 50 | Fe_2_O_3_ | 6.585 | 1,706,504 | 26.04 | 0.95 |
| 7 | 0.01 | 50 | SiO_2_ | 7.108 | 1,881,347 | 26.90 | 0.97 |

**Table S2 Theoretically calculated parameters for ice-water interface energy.**

| Case No. | *α* (vol%) | *D* (nm) | Type | *γ*_gw_ (mJ/m^2^) | *γ*_pg_ (mJ/m^2^) | cos*θ*_int_ | *f*_p_ |
| --- | --- | --- | --- | --- | --- | --- | --- |
| 1 | / | / | / | 72.8 | / | / | / |
| 2 | 0.001 | 50 | TiO_2_ | 72.8 | 600 | 0.766 | 5.61×10^-4^ |
| 3 | 0.01 | 50 | TiO_2_ | 72.8 | 600 | 0.766 | 2.60×10^-3^ |
| 4 | 0.1 | 50 | TiO_2_ | 72.8 | 600 | 0.766 | 1.21×10^-2^ |
| 5 | 0.01 | 50 | Al_2_O_3_ | 72.8 | 1,200 | 0.819 | 2.60×10^-3^ |
| 6 | 0.01 | 50 | Fe_2_O_3_ | 72.8 | 300 | 0.643 | 2.60×10^-3^ |
| 7 | 0.01 | 50 | SiO_2_ | 72.8 | 100 | 0.574 | 2.60×10^-3^ |

Note S3. Theoretical calculation of nucleation temperature in pure water

The nucleation temperature for water solidification can be calculated using the Gibbs nucleation energy barrier. The Gibbs free energy barrier is expressed by

$\Delta G^{*}=\frac{16\text{π}\gamma_{\text{iw}}^{3}T_{\text{f}}^{2}f\left(\theta_{\mathrm{Y}}\right)}{3\Delta H_{\mathrm{V}}^{2}\left(T_{\mathrm{f}}-T_{\mathrm{n}}\right)^{2}}$ **(S14)**

where *γ*_iw_ is the effective interfacial energy between ice and NPW, *T*_f_ and *T*_n_ are the freezing and nucleation temperatures, *f*(*θ*_Y_) is the Fletcher factor related to the Young’s contact angle (*θ*_Y_) of the basement, and Δ*H*_V_ is the volumetric enthalpy of ice melting. Therefore, the nucleation temperature can be calculated as

$T_{\mathrm{n}}=T_{\mathrm{f}}-\sqrt{\frac{16\text{π}\gamma_{\text{iw}}^{3}T_{\text{f}}^{2}f\left(\theta_{\mathrm{Y}}\right)}{3\Delta G^{*}\Delta H_{\mathrm{V}}^{2}}}$ **(S15)**

Note S4. Modeling of dendrite height evolution

At the early stage of nucleation and recalescence, the dendrite growth in water droplet is driven by temperature difference of water. During the dendrite growth process, it is assumed that all the energy released by the growth is considered to be transferred to the water through thermal conductivity. Therefore, the energy conservation is expressed as

$\rho_{\text{ice}}v_{\text{d}}L=-k_{\text{ice}}\left(\frac{\partial T}{\partial H}\right)_{H=0}$ **(S16)**

where *v*_d_ is the dendrite growth velocity, *ρ*_ice_ is the ice density, *k*_ice_ is the thermal conductivity of ice, *L* is the phase-change latent heat, and *∂T*/*∂H* is the gradient in water on the liquid side of the interface. Then, the gradient in the water is calculated based on the boundary layer theory from **Eq. S16**, ^[17]^ i.e.,

$-\left(\frac{\partial T}{\partial H}\right)_{H=0}=\zeta\left(\frac{v_{\text{w}}}{\nu R_{\text{d}}}\right)^{1/2}\left(T_{\text{interface}}-T_{\text{n}}\right)$ **(S17)**

where *ζ* is the dimensionless temperature gradient at the ice-water interface, *v*_w_ is the flow velocity of water, *υ* is the kinematic viscosity of water, *R*_d_ is the curvature radius of the dendrite tip, and *T*_interface_ is the ice-water interface temperature obtained from

$T_{\text{f}}-T_{\text{interface}}=\frac{\gamma_{\text{eff}}T_{\text{f}}}{\rho_{\text{ice}}LR_{\text{d}}}$ **(S18)**

Furthermore,

$T_{\text{interface}}-T_{\text{n}}=T_{\text{f}}-T_{\text{n}}-\left(T_{\text{f}}-T_{\text{interface}}\right)=T_{\text{f}}-T_{\text{n}}-\frac{\gamma_{\text{iw}}T_{\text{f}}}{\rho_{\text{ice}}LR_{\text{d}}}$ **(S19)**

Combining **Eqs. S16 to S19** yields

$v_{\text{d}}=\frac{\zeta k_{\text{ice}}}{\rho_{\text{ice}}L}\left(\frac{v_{\text{w}}}{\nu R_{\text{d}}}\right)^{1/2}\left(T_{\text{f}}-T_{\text{n}}-\frac{\gamma_{\text{iw}}T_{\text{f}}}{\rho_{\text{ice}}LR_{\text{d}}}\right)$ **(S20)**

The *R*_d_ can be calculated by ^[17]^

$R_{\text{d}}=\frac{3\gamma_{\text{iw}}T_{\text{f}}}{\rho_{\text{ice}}L\left(T_{\text{f}}-T_{\text{n}}\right)}$ **(S21)**

Combining **Eqs. S20 and S21** generates

$v_{\text{d}}=\frac{2\zeta k_{\text{ice}}v_{\text{w}}^{1/2}}{\left(27\gamma_{\text{iw}}\rho_{\text{ice}}\upsilon LT_{\text{f}}\right)^{1/2}}\left(T_{\text{f}}-T_{\text{n}}\right)^{3/2}\sim\left(T_{\text{f}}-T_{\text{n}}\right)^{3/2}$ **(S22)**

According to the relationship between the dendrite average height ($\bar{H}_{\text{d}}$) and time, i.e., $\bar{H}_{\text{d}}$ = *v*_d_*t.* Therefore, based on **Eq. S22**, the relationship between the dendrite average height and time could be described as

$\bar{H}_{\mathrm{d}}\sim\left(T_{\mathrm{f}}-T_{\mathrm{n}}\right)^{1.5}\text{t}$ **(S23)**

Note S5. Dendrite height and number density during nucleation and recalescence stage

In the nucleation and recalescence stage, the dendritic growth is driven by the release of latent heat. When the latent heat is transferred to the supercooled liquid via the thermal diffusion, the dendrites grow. The characteristic of thermal diffusion is that diffusion length increases with the square root of time. The diffusion length is defined as

$l_{\mathrm{dif}}=\sqrt{C_{\mathrm{therm}}t}$ **(S24)**

where *C*_therm_ is the thermal diffusion coefficient. The solid-liquid interface at the tip of the dendrite is the core region for latent heat release. The dendrite height, $\bar{H}_{\text{d}}$, is determined by the longitudinal length of latent heat diffusion. Therefore, the dendrite height is proportional to the thermal diffusion length, i.e.,

$\bar{H}_{\mathrm{d}}\sim l_{\mathrm{dif}}=\sqrt{C_{\mathrm{therm}}t}$ **(S25)**

The dendrite gap (*G*) is determined by the interaction of thermal diffusion fields between dendrites. If the dendrite gap is too small, latent heat accumulation causes an increase in interfacial temperature, inhibiting growth. If the dendrite gap is too large, the supercooled liquid is not fully utilized. Therefore, during steady-state growth, the dendrite gap is proportional to the thermal diffusion length, i.e.,

$G\sim l_{\mathrm{dif}}=\sqrt{C_{\mathrm{therm}}t}$ **(S26)**

Combining **Eqs. S25** and **S26** yields

$\bar{H}_{\mathrm{d}}\sim G$ **(S27)**

The number of dendrites per unit length (*n*) can be calculated as *n*=1/*G*. So, the dendrite height exhibits a negative correlation with the dendrite number density, i.e.,

$\bar{H}_{\mathrm{d}}\sim\frac{1}{n}$ **(S28)**

Note S6. Modeling of the tip morphology of solidified droplets

To further elucidate this phenomenon, we established a physical model describing the formation of the pointy tip at the apex of the solidifying droplet. Governed by quasi-static heat conduction, the freezing front of a 2D droplet exhibits a circular arc, as depicted in **Figure S30**. Incorporating the law of conservation of mass allows the time derivative to be mapped onto a derivative with respect to the three-phase contact line height *y*. The relative variation between the liquid and solid areas within the 2D system is thus formulated as follows ^[18]^

$\frac{\text{d}}{\text{d}y}\left(S_{\text{l}}+\varsigma S_{\text{s}}\right)=0$ **(S29)**

where *S*_l_ and *S*_s_ are the respective cross-sectional areas of the liquid and solid phases, and *ς* = *ρ*_s_/*ρ*_l_ is the solid-to-liquid density ratio. The total area of the 2D liquid phase is partitioned into an upward circular segment with a local inclination angle of *β*_2_ and an area *S*_sector_ that protrudes downward into the solid phase. Consequently, the areas of the liquid and solid phases are expressed as

$S_{\text{l}}=R_{\text{f}}g\left(\beta_{2}\right)+S_{\text{sector}}$ **(S30)**

$S_{\mathrm{s}}=-S_{\text{sector}}+\int_{0}^{y} 2R_{\text{f}}\left(y'\right)\mathrm{d}y'$ **(S31)**

where *R*_f_ is the local half-width at the contact line. For a 2D circular segment, the geometric function *g*(*β*_2_) corresponding to the angle *β*_2_ is defined as

$g\left(\beta_{2}\right)=\frac{\beta_{2}-\sin\beta_{2}\cos\beta_{2}}{\sin^{2}\beta_{2}}$ **(S32)**

In the late stage of the freezing process, the freezing front evolves into a downward-concave circular segment, where the downward angle is defined by *β*_3_ = *β*_1_ - *β*_2_. The downward-extended area is thus given by $S_{\text{sector}}=R_{\text{f}}^{2}g\left(\beta_{1}-\beta_{2}\right)$, under the assumption of a constant *β*_1_ = 90°. Recognizing that the geometric variation of the contact-line half-width with height obeys $\text{d}R_{\text{f}}\text{/d}y=-\text{1/tan}\beta_{2}$, differentiation of **Eq. S29** leads to

$\frac{\text{d}\left(R_{\text{f}}^{2}\right)}{\text{d}y}\left[g\left(\beta_{2}\right)+\left(1-\varsigma\right)g\left(\beta_{1}-\beta_{2}\right)\right]+2\varsigma R_{\text{f}}=0$ **(S33)**

Inserting $2R_{\text{f}}=-\text{tan}\beta_{2}\text{d}\left(R_{\text{f}}^{2}\right)/\text{d}y$ into **Eq. S33** leads to

$\frac{\text{d}\left(R_{\text{f}}^{2}\right)}{\text{d}y}\left[g\left(\beta_{2}\right)+g\left(\beta_{1}-\beta_{2}\right)-\varsigma g\left(\beta_{1}-\beta_{2}\right)-\varsigma\tan\beta_{2}\right]=0$ **(S34)**

Nearing the tip singularity where *R*_f_ tends to zero, the term inside the square brackets must be zero to satisfy the equation. Consequently, **Eq. S34** becomes

$g\left(\beta_{2}\right)+g\left(\beta_{1}-\beta_{2}\right)=\varsigma g\left(\beta_{1}-\beta_{2}\right)+\varsigma\tan\beta_{2}$ **(S35)**

With *β*_2_ obtained via **Eq. S35**, the ultimate pointy tip angle is subsequently calculated as $\beta=\text{π}-2\beta_{2}$.

**Figure S30. Schematic illustration of the tip morphology of a solidifying droplet.**

To intuitively reveal the comprehensive influence of nanoparticle parameters on this final macro-morphology, we have further provided localized experimental photographs of the finalized solidified droplet tips under various working conditions in **Figure S31**. As captured in **Figure S31**, the DIW droplet exhibits a classic, pronounced pointy protrusion at its apex. In contrast, for the nanoparticle-containing droplets, the degree of vertical apex protrusion is significantly weakened, causing the corresponding tip angle *β* to expand and widen. Specifically, under a fixed particle diameter of 50 nm and type of TiO_2_, increasing the nanoparticle concentration from 0 to 0.01 vol% causes the frozen tip profile to transition progressively from a sharp singularity to a smooth, flat arc. This directly demonstrates that a higher concentration of nanoparticles exerts a powerful inhibitory effect on the formation of the solidification apex singularity. By implementing our 2D theoretical tip model, we successfully quantified the relationship between these macro-morphological variations and the underlying microphysical parameters. The analytical calculations reveal that as the tip angle *β* gradually expands from 136° in pure water to 170° under TiO_2_-α2-D50, the corresponding local effective density ratio *ς* continuously elevates from 0.89 to 0.98. Remarkably, beyond a certain threshold of tip blunting, the computed local effective density ratio surpasses the density ratio of a pure ice-water system (*ς* = 0.92).

**Figure S31. Photograph of the tip morphology of a solidified droplet.** The scale bar is 0.3 mm.

This prominent elevation in the localized effective density ratio is governed by a dual physical mechanism involving bubble suppression and particle segregation. (1) In pure water or low-concentration suspensions, the fast overall freezing rate causes dissolved air expelled at the freezing front to be trapped rapidly before escaping ^[19,20]^, creating a vast population of microscopic trapped air bubbles as observed in **Figure S31**. These low-density bubbles inevitably reduce the effective density of the solid phase region. Increasing the nanoparticle concentration markedly retards the advancement velocity of the freezing front, granting ample time for gas diffusion and escape. This severe suppression of trapped bubbles inherently drives up the density of the solid phase. (2) A highly unique nanoparticle enrichment effect occurs at the droplet apex. Driven by the slowed freezing kinetics, the solid-liquid interface exhibits a more pronounced rejection effect against the suspended phase, continuously segregating and pushing high-density solid nanoparticles toward the apex of the droplet ^[21-23]^. This continuous frontal pushing increases the local solid particle volume fraction at the final stage of solidification, thereby boosting the localized effective density ratio well beyond the conventional baseline of pure water.

Note S7. Thermodynamic correlation between 2D and 3D droplet solidification

The fundamental physical justification for utilizing the 2D Hele-Shaw cell configuration (2 μL droplet confined within a 0.3 mm spacer gap) is that it serves as an excellent thermodynamic and kinetic analogue to real 3D systems while resolving a severe optical bottleneck. In 3D droplet solidification, the curved, unconfined hemispherical boundary and complex light scattering at the advancing phase front severely hamper high-resolution, in situ visual observations of internal transient features, rendering microstructural ice dendrite growth and bubble nucleation behaviors hazy or practically invisible.

Despite the geometric difference, both 2D and 3D droplets progress through the exact same four sequential stages of solidification: supercooling, nucleation and recalescence, freezing, and cooling. The underlying physical mechanisms through which nanoparticles modulate droplet solidification, namely, lowering the heterogeneous nucleation energy barrier, densifying the primary dendritic network, and retarding the local freezing front via liquid thermal conductivity enhancement, are identical universal transport phenomena operating in both configurations. The primary variations between the two systems lie in the dimensionality of heat/mass transfer and the absolute scales of volume-dependent properties. In the 2D cell, heat transfer is simplified to a near-planar, directional flow along the vertical axis, whereas a 3D droplet experiences multi-directional heat dissipation into both the cold plate and the surrounding environment. This dimensionality shift, combined with the larger absolute volume-to-surface-area ratio of 3D droplets, leads to minor variations in solidification timescales and nucleation temperatures (*T*_n_). Specifically, the larger supercooled bulk volume of 3D droplets inherently increases the statistical probability of capturing active heterogeneous nucleation sites, shifting their *T*_n_ slightly higher compared to their 2D counterparts. However, because the fundamental scaling laws governing gas diffusion dynamics and mass conservation remain invariant to these scale shifts, the microscopic bubble suppression mechanisms validated in our 2D visualization platform support transfer to the mechanical reinforcement of printed 3D structural components.

Note S8. Modeling of freezing front height with time during the freezing stage

As shown in **Figure S32**, in the freezing stage of a 2D droplet, the phase-change latent heat released by solidification is mainly transferred through the cold plate and the boundary, so the energy conservation is expressed as

$\rho_{\text{l}}aL\text{d}H_{\text{f}}=ak_{\text{ice}}\frac{T_{\text{f}}-T_{\text{c}}}{H_{\text{f}}}\text{d}t-2ah_{\text{air}}H_{\text{f}}\left(T_{\text{air}}-T_{\text{cell}}\right)\text{d}t-ah_{\text{l}}\left(T_{\text{l}}-T_{\text{f}}\right)\text{d}t$ **(S36)**

where *a* is the gap width of the Hele-Shaw cell, *T*_f_, *T*_air_, *T*_cell_, and *T*_l_ are the freezing temperature, air temperature, cell temperature, and the average temperature of the liquid droplet during freezing, *ρ*_l_ is the density of liquid droplets, *k*_ice_ is the thermal conductivity of ice, *h*_air_ and *h*_l_ are the heat transfer coefficients between the cell and air and between ice and liquid droplet at the freezing front. For laminar natural convection over a downward-cooling surface, the heat transfer coefficient could be estimated by

$\frac{\text{h}_{\mathrm{l}}l}{k_{\mathrm{l}}}=0.54Ra_{l}^{0.25}$ **(S37)**

where *k*_l_ is the thermal conductivity of liquid droplet, *Ra* is the Rayleigh Number, i.e.,

$Ra_{l}=\frac{\eta g\left(T_{\text{l}}-T_{\text{f}}\right)l^{3}}{\nu^{2}}\frac{\mu c_{p}}{k_{\text{l}}}$ **(S38)**

where *g* is the acceleration of gravity, *η* is the coefficient of thermal expansion, *l* is the characteristic dimension, *μ* is the dynamic viscosity, *ν* is the kinematic viscosity. Therefore, **Eq. S37** could be described as

$\frac{\text{h}_{\mathrm{l}}l}{k_{\mathrm{l}}}=0.54\left[\frac{\eta g\left(T_{\mathrm{l}}-T_{\mathrm{f}}\right)l^{3}}{\nu^{2}}\frac{\mu c_{p}}{k_{\text{l}}}\right]^{0.25}$ **(S39)**

The *h*_l_ could be calculated as

$\text{h}_{\mathrm{l}}=0.54\left[\frac{\mu\eta c_{p}g\left(T_{\mathrm{l}}-T_{\mathrm{f}}\right)}{\nu^{2}\text{l}}\right]^{0.25}k_{\mathrm{l}}^{0.75}\sim k_{\mathrm{l}}^{0.75}$ **(S40)**

According to the thermal conductivity of NPW droplets, the relationship between the heat transfer coefficient between ice and liquid could be scaled as *h*_l_ = 835*k*_l_^0.75^ (**Figure S33**).

**Figure S32. Schematic diagram of the heat transfer mechanism in the 2D droplet during freezing stage. (A) Front view and (B) Side view.**

**Figure S33. Change of the thermal conductivity of liquid droplet with the heat transfer coefficient between the freezing front and liquid droplet.**

Note S9. Temperature distribution inside the droplets during the supercooling stage

The droplets do not nucleate immediately after the cold plate is cooled down to the set temperature, but there is a period of supercooling of the droplets. The average supercooling times of DIW, SiO_2_-α2-D50, TiO_2_-α2-D50, and TiO_2_-α2-D20 droplets are 65 s, 57 s, 50 s, and 42 s. The temperature distribution inside droplets is directly affected by the subcooling time, so we use ANSYS Fluent software to simulate the temperature distribution inside droplets during subcooling. **Figure S34** shows the fluid domain and the structure domain that we have modelled. Firstly, we built the geometric model based on the actual dimensions of the Hele-Shaw cell and droplets. The initial temperature (*T*_0_) of the 2D droplets is 20 °C and the cold plate temperature is -20 °C. To prevent condensation fog on the surface of the Hele-Shaw cell during the experiment, we make an environmental chamber that can wrap around the Hele-Shaw cell and continuously fill it with nitrogen, so there is a weak convection heat transfer between the nitrogen and the cell. Therefore, we set a heat transfer coefficient (*h*_air_) of 12 W/(m^2^·K) slightly larger than the natural convection heat transfer coefficient. The air temperature is set to 20 °C. The results show that the temperature at the top of the DIW droplet (*T*_top_) is still above zero after 65 s of supercooling. For other droplets, the temperatures at their tops are larger due to the shorter supercooling time. The temperatures at the top of the DIW, SiO_2_-α2-D50, TiO_2_-α2-D50, and TiO_2_-α2-D20 droplets are 1.7 ℃, 2.6 ℃, 3.4 ℃, and 4.5 ℃, and their average is 3 ℃. So, we set the initial temperatures at the top of all droplets (*T*_top,0_) to 3 ℃. Then, we assume the change of droplet top temperature is linear during the freezing stage, i.e., *T*_top_ ~ (*H*_droplet_ - *H*_f_)/*H*_droplet_, the temperature of the liquid droplet (*T*_l_) can be described as

$T_{\mathrm{l}}=\frac{T_{\mathrm{top},0}\left(H_{\mathrm{droplet}}-H_{\mathrm{f}}\right)+T_{\mathrm{f}}H_{\mathrm{droplet}}}{2H_{\mathrm{droplet}}}$ **(S41)**

where *H*_dropet_ is the initial height of droplets, *H*_f_ is the freezing front height, and *T*_f_ is the freezing temperature.

**Figure S34. Temperature distribution inside 2D DIW and NPW droplets during supercooling stage.**

**(A)** Computational domain.

**(B)** Temperature distribution of 2D droplets. The scale bar is 1 mm.

Note S10. Thermal conductivity of nanoparticle suspension

Due to the interactions between the nanoparticles and liquid molecules, the liquid molecules adsorb in an ordered manner on the surface of nanoparticles to form an adsorption layer. ^[24]^ The nanoparticles with adsorption layers are considered as composite nanoparticles. Then, the volume fraction of composite nanoparticles (*α*_comp_) is calculated by

$\alpha_{\mathrm{comp}}=\left(1+\frac{2\delta}{D}\right)^{3}\alpha$ **(S42)**

where *D* is the nanoparticle diameter, *α* is the nanoparticle concentration, *δ* is the thickness of the adsorption layer, *δ* = (2π)^0.5^*ψ*, and the value of *ψ* is 0.2-0.8 nm. The thermal conductivity of composite nanoparticles (*k*_comp_) can be calculated by the improved Maxwell model, ^[25]^, i.e.,

**(S43)**

where *k*_p_ and *k*_layer_ are the thermal conductivities of the nanoparticle and adsorption layer, and *k*_layer_ can be calculated by

**(S44)**

where *C*_1_ = *C*_4_/(*k*_p_+*C*_4_*D*/2)^2^, *C*_2_ = 1/(*k*_p_+*C*_4_*D*/2), *C*_3_ = [*C*_4_/(*k*_p_+*C*_4_*D*/2)]^2^, and *C*_4_ = (*k*_p_-*k*_fluid_)/*δ*, *k*_fluid_ is the thermal conductivity of dispersion fluid. Considering the combined effects of the adsorption layer, particle agglomeration, and Brownian movement, the thermal conductivity of nanoparticle suspension (*k*_l_) can be calculated by ^[26]^

$\frac{\text{k}_{\mathrm{l}}}{k_{\mathrm{fluid}}}=\left(1+C'\text{Re}^{\mathrm{m}}\text{Pr}^{0.333}\alpha_{\mathrm{comp}}\right)\left[\frac{k_{\mathrm{comp}}+2k_{\mathrm{fluid}}+2\alpha_{\mathrm{comp}}\left(k_{\mathrm{comp}}-k_{\mathrm{fluid}}\right)}{k_{\mathrm{comp}}+2k_{\mathrm{fluid}}-\alpha_{\mathrm{comp}}\left(k_{\mathrm{comp}}-k_{\mathrm{fluid}}\right)}\right]$ **(S45)**

where *C*’ and *m* are the empirical coefficients, *C*’ = 4,000, *m* is related to the fluid, *m*=1.9, *Pr* is the Prandtl number, *Pr* = *μc_p_*/*k*_fluid_, *μ* is the dynamic viscosity, *c_p_* is the specific heat capacity, and *Re* is the Reynolds number. Considering the fluid convection near the nanoparticle caused by Brownian movement of nanoparticles, the Reynolds number can be written as

$\text{Re}=\frac{1}{\mu}\sqrt{\frac{18\lambda_{\mathrm{B}}T}{\pi\rho_{\mathrm{p}}D}}$ **(S46)**

where *λ*_B_ is the Boltzmann constant, *ρ*_p_ is the nanoparticle density. The thermal conductivity of NPW droplets calculated by **Eq. S45** at various nanoparticle concentrations, diameters, and types is shown in **Figure S35**. The thermal conductivity of Al_2_O_3_, TiO_2_, Fe_2_O_3_, and SiO_2_ nanoparticles are 30 W/(m·K), 7.9 W/(m·K), 11.8 W/(m·K), and 1.4 W/(m·K) ^[27-30]^. The densities of Al_2_O_3_, TiO_2_, Fe_2_O_3_, and SiO_2_ nanoparticles are 3.79 g/cm^3^, 4.26 g/cm^3^, 5.24 g/cm^3^, and 2.7 g/cm^3^ ^[31-34]^.

**Figure S35. Variations of thermal conductivity with nanoparticle concentration and diameter for (A) Al_2_O_3_, (B) TiO_2_, (C) Fe_2_O_3_, and (D) SiO_2_ NPW droplets.**

Note S11. Modeling of bubble width of trapped air bubbles with freezing rate

Bubble width is the maximum value attainable by the bubble in the direction perpendicular to the freezing direction during its expansion process. The bubble volume increase in water is related to the air diffusion, which can be described by ^[35]^

$\frac{\mathrm{d}V_{\mathrm{b}-\mathrm{w}}}{\mathrm{d}t}=-A_{\mathrm{b}}C_{\mathrm{dif}}\left(\frac{\partial c}{\partial R_{\mathrm{b}}}\right)_{R_{\mathrm{b}}}\frac{M_{\mathrm{air}}\rho_{\mathrm{w}}}{\rho_{\mathrm{air}}}$ **(S47)**

where *V*_b-w_ is the bubble volume in water, *A*_b_ is the area of the bubble-water interface, *C*_dif_ is the diffusion coefficient of gas in water, *c* is the concentration of surrounding air, *R*_b_ is the bubble radius, *M*_air_ is the molar mass of air, *ρ*_w_ and *ρ*_air_ are the densities of water and air, *∂c*/*∂R*_b_ is the concentration gradient of dissolved air at the gas-water interface of the bubble. When the air concentration near the bubble reaches steady state, the concentration gradient can be expressed by ^[36]^

$\left(\frac{\partial c}{\partial R_{\mathrm{b}}}\right)_{R_{\mathrm{b}}}\approx C_{0}\frac{c_{\infty}-c_{0}}{R_{\mathrm{b}}}$ **(S48)**

where *C*_0_ is the correction factor related to the freezing process, *c*_∞_ is the initial concentration of air dissolved in water, and *c*_0_ is the dissolved air concentration in water at saturation. Due to the little solubility of air in water, the air concentration in water can be calculated by Henry’s law, i.e., ^[37]^

$c_{\infty}-c_{0}=\frac{\Delta P}{C_{\mathrm{H}}}$ **(S49)**

where Δ*P* is the pressure difference between the inside and outside of the bubble, and *C*_H_ is the Henry’ law constant. The shape of a bubble expanding in water is hemispherical and can be described by the Young-Laplace equation ^[38]^, i.e.,

$\Delta P=\frac{2\kappa}{R_{\mathrm{b}}}$ **(S50)**

where *κ* is the surface tension of the liquid. The volume change rate of a bubble in water can be expressed as

$\mathrm{d}V_{\mathrm{b}-\mathrm{w}}\text{=}\pi R_{\mathrm{b}}^{2}\mathrm{d}R_{\mathrm{b}}$ **(S51)**

Combining **Eqs. S47 to S51** generates

$W_{\mathrm{b}}=\sqrt{\frac{6C_{\mathrm{dif}}C_{0}M_{\mathrm{air}}\rho_{\mathrm{w}}\kappa}{\rho_{\mathrm{air}}C_{\mathrm{H}}}\frac{t}{W_{\mathrm{b}}}}$ **(S52)**

Assuming bubble growth can be considered quasi-steady state, *v*_f_ = *W*_b_/*t* can be regarded as the freezing rate. **Eq. S52** is expressed as

$W_{\mathrm{b}}=\sqrt{\frac{6C_{\mathrm{dif}}C_{0}M_{\mathrm{air}}\rho_{\mathrm{w}}\kappa}{\rho_{\mathrm{air}}C_{\mathrm{H}}}}\frac{1}{\sqrt{\text{v}_{\mathrm{f}}}}\sim\frac{1}{\sqrt{\text{v}_{\mathrm{f}}}}$ **(S53)**

As shown in **Figure S36**, this universal data collapse demonstrates that the power law is fundamentally governed by pure mass transport across the localized concentration boundary layer, independent of nanoparticle surface chemistry. Because the key transport parameters dictating this scaling relation, including the gas diffusion coefficient, Henry’s law constant, and bulk surface tension, are intrinsic thermodynamic properties of the base water-air system, the bubble growth kinetics decouple from the chemical nature of the additives once continuous macroscopic freezing is established. Rather than altering the intrinsic gas diffusion mechanism itself, the nanoparticles merely modulate the absolute size of the trapped air bubbles indirectly by shifting the local freezing rate (*v*_f_). Furthermore, the equivalent diameter and number of bubbles are used to confirm that the influence of nanoparticles on bubble formation is primarily mediated through their effect on the freezing rate of the droplet (**Figure S37**).

**Figure S36. Changes of bubble widths with freezing rate under various nanoparticle (A) concentrations, (B) diameters, and (C) types.**

**Figure S37. Changes of (A) bubble equivalent diameters and (B) bubble number with freezing rate under various nanoparticle concentrations.**

Note S12. Modeling of volume fraction of trapped air bubbles in solidified droplet

During water freezing, a large number of gas molecules are expelled and accumulate near the freezing front ^[39,40]^. Simultaneously, the concentrated gas diffuses into the surrounding liquid. To quantitatively describe the resulting gas concentration distribution, Carte modeled the diffusion process as a one-dimensional unsteady problem and expressed the local gas concentration as ^[35,41]^

$\frac{\partial\text{c}}{\partial t}=C_{\mathrm{dif}}\frac{\partial^{2}\text{c}}{\partial x^{2}}+\frac{\mathrm{d}H_{\mathrm{f}}}{\mathrm{d}t}\frac{\partial\text{c}}{\partial x}$ **(S54)**

where *c* is the gas concentration, *C*_dif_ is the diffusion coefficient of gas in water, *x* is the distance from a point ahead of the freezing front to the freezing front, and taking *x* as the average distance between the freezing front and the droplet apex yields *x* = (*H*_droplet_-*H*_f_)/2. Substituting *c* = *c*_0_ for the initial condition *t* = 0 into the above equation yields

$\text{c}_{\mathrm{air}}\text{=c}_{0}\left\{1+\frac{1-C_{\mathrm{dis}}}{C_{\mathrm{dis}}}\left[e^{-\frac{x\text{v}_{\mathrm{f}}}{C_{\mathrm{dif}}}}-e^{-\frac{\text{v}_{\mathrm{f}}\left(1-C_{\mathrm{dis}}\right)\left(x+C_{\mathrm{dis}}\text{v}_{\mathrm{f}}t\right)}{C_{\mathrm{dif}}}}\right]\right\}$ **(S55)**

where *C*_dis_ is the distribution coefficient, which is defined as the ratio of the concentrations of solute at the interface in the solid and in the liquid, respectively, *c*_0_ is the initial gas concentration, and *v*_f_ is the transient freezing rate. For the volume fraction of trapped air bubble in ice, we assume the freezing front is flat and the bubbles are incompressible. After an infinitesimal time interval, d*t*, the freezing front advances by d*H*_f_, and the volume of bubbles released, d*V*_b_, given by

$\mathrm{d}\sum V_{\mathrm{b}}\approx2\text{apR}_{\mathrm{f}}\mathrm{d}\text{H}_{\mathrm{f}}$ **(S56)**

where *p* is the volume fraction of trapped air bubbles in ice, *a* is the gap width of the Hele-Shaw cell, *C*_air_ is a correction factor used to account for gases expelled from droplets into the air during the solidification process, and *R*_f_ is the radius of the freezing front. Considering the volume conservation of water and bubbles, the bubble volume fraction is related to the air solubility in water, i.e., *c*_air_ = *p*/[*ς*(1−*p*)], where *ς* is the ratio of ice and water densities, Therefore, the volume of bubbles released within time d*t* is given by

$\mathrm{d}\sum V_{\mathrm{b}}\approx2\left(1+\frac{1}{\varsigma\text{c}_{\mathrm{air}}}\right)^{-1}\text{aC}_{\mathrm{air}}\text{R}_{\mathrm{f}}\mathrm{d}\text{H}_{\mathrm{f}}$ **(S57)**

The relationship between the freezing front radius and the freezing front height is expressed as

$R_{\text{f}}=\sqrt{R_{0}^{2}-\left(H_{\text{f}}+R_{0}\cos\theta_{\text{droplet}}\right)^{2}}$ **(S58)**

where the *R*_0_ is the initial radius of the droplet, and *θ*_droplet_ is the contact angle between the droplet and cold plate. Combining **Eqs. S57 and S58**, the ratio of bubble change is derived as

**(S59)**

So, the total volume of trapped air bubbles in ice can be obtained by combining **Eqs. S36 and S59**. Finally, the volume fraction of trapped air bubbles in the solidified droplet is calculated as

$\text{p}=\frac{\varsigma V_{\mathrm{b}}}{\varsigma V_{\mathrm{b}}+V_{\mathrm{droplet}}}$ **(S60)**

where *V*_droplet_ is the volume of the liquid droplet.

Note S13. Numerical simulation of mechanical strength of solidified droplets

Numerical simulations of compressive stress in solidified droplets are conducted using the ANSYS Static Structural. A geometric model for computational purposes is constructed based on the actual contour shape of the solidified droplet and the internal bubble distribution, as shown in **Figure S38.** The droplet thickness is 0.3 mm. The contact angle between the solidified droplet and the substrate is 140°. All bubbles are centered along the thickness direction. During experimental compression testing of solidified droplets, despite our efforts to minimize thermal effects from the room-temperature pressure sensor, slight melting still occurs at the droplet apex. To accurately represent this phenomenon, the geometric model incorporates a flattened surface at the droplet tip, corresponding to the actual melted region observed in mechanical tests. In the mechanical compression simulation of solidified droplets, the elastic modulus is set to 10 GPa, the Poisson’s ratio is set to 0.33, and the density *ρ*_ice_ is set to 920 kg/m^3^.

Meshing is performed within the ANSYS Mechanical module using an automatic meshing scheme with tetrahedral elements specified. Local mesh refinement is applied around the bubble regions inside the droplet. For the solidified DIW droplets, through mesh density enhancement and comparison of maximum equivalent stress values as shown on **Table S3**, we determine that employing approximately 485,454 elements yielded a relative deviation of less than 5% upon further refinement, thus confirming the attainment of a grid-independent solution. For the boundary condition setup: (1) A fixed constraint is applied to the bottom surface where the droplet contacts the substrate, and (2) A fixed force load is applied to the top surface of the droplet. The Static Structural solver in ANSYS Mechanical is selected for the solution.

Following the application of a 1 N force at the apex of the solidified droplet, the resulting distributions of equivalent stress and tensile stress are shown in **Figure S39**. The results indicate that the primary cause of failure under compressive loading is the development of internal tensile stresses. To validate the accuracy of these mechanical simulations, we subsequently perform tensile mechanical experiments on ice specimens. The ice specimen has a diameter of 50 mm and a thickness of 30 mm. To accurately obtain the permissible tensile strength (*σ*_t_) of ice and avoid interference from internal bubble defects, clear ice is selected as the experimental material in this study. Experiments are conducted on disk-shaped ice specimens with a diameter (*D*_ice_) of 50 mm and thickness (*H*_ice_) of 30 mm, under a constant ambient temperature of -20 °C. Under compression, the specimen fails at a load *F*. The permissible tensile strength is derived from the analytical solution for the Brazilian test, given by ^[42]^

$\sigma_{\text{t}}=\frac{2F}{\text{π}D_{\text{ice}}H_{\text{ice}}}$ **(S61)**

Concurrently, a numerical model of the ice specimen is developed based on its geometric dimensions. Mechanical simulations are performed to analyze the tensile stress distribution characteristics across its longitudinal section, with results present in **Figure S39**. To validate the reliability of the simulation methodology, the computationally predicted tensile strength is compared against experimentally measured values. As summarized in **Table S4**, the discrepancy between the two sets of results is less than 20%, confirming that the simulation model reasonably captures the tensile mechanical behavior of ice.

The maximum compressive strength of the solidified 2D droplets is defined by the permissible tensile strength of ice (*σ*_t_). Based on our experiments and literature data, *σ*_t_ is set to 1 MPa ^[43]^_._ A force is applied at the droplet apex until the maximum internal tensile stress reaches this threshold, with the corresponding critical force used to calculate the maximum compressive strength as *σ*_max_ = *F*_max_/*A*, where *A* represents the contact area between the solidified droplets and the cold plate.

**Figure S38. Simulation of mechanical performance of solidified droplets.**

**(A)** Geometric model.

**(B)** Mesh grids.

**(C)** Equivalent stress contour map.

**(D)** Tensile stress contour map. The scale bar in full and magnified views are 1 mm, 0.5 mm and 0.02 mm.

**Figure S39. Experiments and simulations of tensile stress of disk-shaped ice.**

**(A)** Experimental images of tensile stress tests of disk-shaped ice. The scale bar is 10 mm.

**(B)** Geometric model of disk-shaped ice.

**(C)** Tensile stress distribution across its longitudinal section.

**Table S3 Mesh independence check.**

| Case No. | *F* (N) | Mesh number | *σ*_max_ (MPa) | Relative error (%) |
| --- | --- | --- | --- | --- |
| 1 | 1 | 289,291 | 5.92 | \ |
| 2 | 1 | 405,784 | 6.59 | 11.31 |
| 3 | 1 | 485,454 | 6.75 | 2.43 |
| 4 | 1 | 613,952 | 6.84 | 1.33 |

**Table S4 Comparison of tensile strength between simulations and experiments.**

| Case No. | *F* (N) | | *σ*_t_ (MPa) | | Relative error (%) |
| --- | --- | --- | --- | --- | --- |
|  | Experiment | Simulation | Experiment | Simulation |  |
| 1 | 1,813 | 2,097 | 0.77 | 0.89 | 15.58 |
| 2 | 1,648 | 1,909 | 0.70 | 0.81 | 15.71 |
| 3 | 2,237 | 2,545 | 0.95 | 1.08 | 13.68 |

Note S14. Calculation of comprehensive evaluation indicators

The viscosity (*μ*) of the nanoparticle suspension, incorporating the effects of base fluid viscosity (*μ*_fluid_), nanoparticle concentration (*α*), diameter (*D*), density (*ρ*_p_), and temperature (*T*) is given by ^[44]^

$\mu=\mu_{\text{fluid}}+\frac{\rho_{\text{p}}V_{\text{p}}D^{2}}{72\alpha C_{\text{v}}}$ **(S62)**

in which

$V_{\text{p}}=\frac{1}{D}\sqrt{\frac{18\lambda_{\text{b}}T}{\text{π}\rho_{\text{p}}D}}$ **(S63)**

$C_{\text{v}}=\mu_{\text{fluid}}^{-1}\left[\left(C_{5}D+C_{6}\right)\alpha+\left(C_{7}D+C_{8}\right)\right]$ **(S64)**

where *λ*_b_ is the Boltzmann constant, *C*_5_, *C*_6_, *C*_7_, and *C*_8_ are constants with values -1.133×10^-6^, -2.771×10^-6^, 9×10^-8^, and 3.39×10^-7^ respectively ^[44]^.

This analytical framework highlights a fundamental divergence in hydrodynamic flow behavior between conventional polymer additives and the present nanoparticle strategy, arising primarily from their contrasting molecular structures and relaxation mechanics under shear. Conventional polymer additives are typically composed of long-chain, flexible macromolecules that undergo extensive chain entanglement and exhibit pronounced viscoelastic memory even at low mass fractions. Under the high shear rates experienced within a drop-on-demand printing nozzle, these polymer chains stretch and uncoil, inducing significant non-Newtonian behaviors such as shear-thinning and normal stress differences. These viscoelastic responses inevitably lead to severe die swell, capillary jet breakup issues, and localized flow stagnation that directly triggers nozzle clogging. In sharp contrast, the rigid nanoparticles used herein operate as isolated solid spheres that completely lack macromolecular uncoiling or elastic relaxation mechanisms, thereby preserving a stable, clean Newtonian flow profile during high-shear extrusion.

To quantify why the formulation escapes these hydrodynamic penalties at the optimized ultra-low concentration of 0.01 vol%, the suspension rheology can be evaluated via classical hydrodynamic transport theory. At such extreme dilution, the average interparticle distance remains significantly larger than the nanometer scale of the particles themselves. This extensive spatial separation effectively suppresses particle-particle hydrodynamic interactions, electrostatic agglomeration, or localized network clustering within printing nozzles whose physical dimensions are several orders of magnitude larger, fundamentally preventing microstructural blockages. Consequently, according to **Eq. S62**, the calculated theoretical viscosity increment is a mere ~ 2.7%. This minimal alteration represents a mathematically and physically negligible hydrodynamic penalty that easily falls within standard experimental margins of error, exerting virtually zero impact on the baseline fluid flowability.

On the other hand, the cost (*c*) of the nanoparticle suspension is evaluated based on the monetary expenditure required for the nanoparticles used in its formulation. The values of the comprehensive impact factor [$g\left(\tilde{D}\right)\tilde{\gamma}_{\text{iw}}^{3}$], conductivity (*k*_l_), viscosity (*μ*), cost (*c*), and the maximum compressive strength (*σ*_max_) of the solidified droplets for DIW, SiO_2_-α2-D50, TiO_2_-α2-D50, and TiO_2_-α2-D20 are provided in **Table S5**.

**Table S5 Specific values for each evaluation criterion.**

| Case | $g\left(\tilde{D}\right)\tilde{\gamma}_{\text{iw}}^{3}$ | *k*_l_  [W/(m·K)] | *σ*_max_ (MPa) | *μ*  (mPa·s) | *c* (RMB/100mL) |
| --- | --- | --- | --- | --- | --- |
| DIW | 1 | 0.6 | 1.398 | 1 | 0 |
| SiO_2_-α2-D50 | 0.945 | 0.613 | 1.446 | 1.0324 | 0.0057 |
| TiO_2_-α2-D50 | 0.850 | 0.617 | 1.460 | 1.0271 | 0.0134 |
| TiO_2_-α2-D20 | 0.613 | 0.648 | 1.551 | 1.0297 | 0.0146 |

**Video S1**

**Name:** Video S1 - Solidification process of 2D and 3D DIW droplets

**Description:** The video shows the difference in the solidification process between 2D and 3D droplets. The video is recorded at 50 FPS and played back at 2.5× speed. The volumes of 3D and 2D droplets are 10 μL and 2 μL. The cold plate temperature is -20 °C, and the air temperature is 20 °C.

**Video S2**

**Name:** Video S2 - Nucleation and recalescence process of DIW and NPW droplets

**Description:** The video shows the difference in dendrite structure between DIW and NPW droplets. The video is recorded at 1,000 FPS and played back at 0.015× speed. The NPW droplet contains TiO_2_ nanoparticles with 0.01% volume concentration and 50 nm diameter. The volumes of DIW and NPW droplets are 2 μL. The cold plate temperature is -20 °C, and the air temperature is 20 °C.

**Video S3**

**Name:** Video S3 - Freezing process of DIW and NPW droplets

**Description:** The video shows the difference in the freezing process between DIW and NPW droplets. The video is recorded at 50 FPS and played back at 4.5× speed. The NPW droplet contains TiO_2_ nanoparticles with 0.01 % volume concentration and 50 nm diameter. The volumes of DIW and NPW droplets are 2 μL. The cold plate temperature is -20 °C, and the air temperature is 20 °C.

**Video S4**

**Name:** Video S4 - Mechanical compression tests of multiple formed DIW and NPW droplets stacked horizontally

**Description:** The video shows the mechanical compression tests of multiple formed DIW and NPW droplets stacked horizontally. The video is recorded at 50 FPS and played back at 2× speed. The NPW droplets contain TiO_2_ nanoparticles with 0.01% volume concentration and 50 nm diameter. The volume of a single droplet is 5 μL. The cold plate temperature is -20 °C, and the air temperature is 20 °C. The loading rate of the push rod is 0.16 mm/s.

**Video S5**

**Name:** Video S5 - Mechanical compression tests of multiple formed DIW and NPW droplets stacked vertically

**Description:** The video shows the mechanical compression tests of multiple formed DIW and NPW droplets stacked vertically. The video is recorded at 50 FPS and played back at 3× speed. The NPW droplets contain TiO_2_ nanoparticles with 0.01% volume concentration and 50 nm diameter. The volume of a single droplet is 5 μL. The cold plate temperature is -20 °C, and the air temperature is 20 °C. The loading rate of the push rod is 0.16 mm/s.

**Video S6**

**Name:** Video S6 - Mechanical compression tests of formed patterns with DIW and NPW droplets

**Description:** The video shows the mechanical compression tests of formed patterns with DIW and NPW droplets. The NPW droplets contain TiO_2_ nanoparticles with 0.01% volume concentration and 50 nm diameter. The volumes of DIW and NPW droplets are 5 μL. The cold plate temperature is -20 °C, and the air temperature is 20 °C. The loading rate of the push rod is 0.16 mm/s.

# References

[1] Abdelmonem, A., Backus, E.H.G., Hoffmann, N., Sánchez, M.A., Cyran, J.D., Kiselev, A. and Bonn, M. Surface-charge-induced orientation of interfacial water suppresses heterogeneous ice nucleation on α-alumina (0001). *Atmos. Chem. Phys.* 17, 7827-7837 (2017). https://doi.org/10.5194/acp-17-7827-2017.
[2] Pedevilla, P., Fitzner, M. and Michaelides, A. What makes a good descriptor for heterogeneous ice nucleation on OH-patterned surfaces. *Phys. Rev. B* 96, 115441 (2017). https://doi.org/10.1103/PhysRevB.96.115441.
[3] Fitzner, M., Pedevilla, P. and Michaelides, A. Predicting heterogeneous ice nucleation with a data-driven approach. *Nat. Commun.* 11, 4777 (2020). https://doi.org/10.1038/s41467-020-18605-3.
[4] Shao, M., Zhang, C., Qi, C., Wang, C., Wang, J., Ye, F. and Zhou, X. Hydrogen polarity of interfacial water regulates heterogeneous ice nucleation. *Phys. Chem. Chem. Phys.* 22, 258-264 (2020). https://doi.org/10.1039/C9CP04867G.
[5] Seveno, D., Blake, T.D. and De Coninck, J. Young’s equation at the nanoscale. *Phys. Rev. Lett.* 2013, 111, 096101. https://doi.org/10.1103/PhysRevLett.111.096101.
[6] Wang, R., Zou, Y., Remsing, R.C., Ross, N.O., Klein, M.L., Carnevale, V. and Borguet, E. Superhydrophilicity of α-alumina surfaces results from tight binding of interfacial waters to specific aluminols. *J. Colloid Interface Sci.* 628, 943-954 (2022). https://doi.org/10.1016/j.jcis.2022.07.164.
[7] Karunakaran, G., Suriyaprabha, R., Rajendran, V. and Kannan, N. Effect of contact angle, zeta potential and particles size on the in vitro studies of Al_2_O_3_ and SiO_2_ nanoparticles. *IET Nanobiotechnol.* 9(1), 27-34 (2015). https://doi.org/10.1049/iet-nbt.2013.0067.
[8] Chen, J.P., Hazra, A. and Levin, Z. Parameterizing ice nucleation rates using contact angle and activation energy derived from laboratory data. *Atmos. Chem. Phys.* 8, 7431-7449 (2008). https://doi.org/10.5194/acp-8-7431-2008.
[9] Gaigeot, M.P., Sprik, M. and Sulpizi, M. Oxide/water interfaces: how the surface chemistry modifies interfacial water properties. *J. Phys.: Condens. Matter* 24, 124106 (2012). https://doi.org/10.1088/0953-8984/24/12/124106.
[10] Zhang, Z.S. and Liu, X.Y. Control of ice nucleation: freezing and antifreeze strategies. *Chem. Soc. Rev.* 47, 7116-7139 (2018). https://doi.org/10.1039/C8CS00626A.
[11] Murray, B.J., O’Sullivan, D., Atkinson, J.D., and Webb, M.E. Ice nucleation by particles immersed in supercooled cloud droplets. *Chem. Soc. Rev.* 41, 6519-6554 (2012). https://doi.org/10.1039/C2CS35200A.
[12] Seidler, G.T. and Seeley, L.H. Two-dimensional nucleation of ice from supercooled water. *Phys. Rev. Let.* 87, 55702 (2001). https://doi.org/10.1103/PhysRevLett.87.055702.
[13] Kelton, K. and Greer, A.L. Nucleation in condensed matter: applications in materials and biology. Amsterdam: Elsevier (2010).
[14] Fu, Q.T., Liu, E.J., Wilson, P. and Chen, Z. Ice nucleation behaviour on sol-gel coatings with different surface energy and roughness. *Phys. Chem. Chem. Phys.* 17, 21492-21500 (2015). https://doi.org/10.1039/C5CP03243A.
[15] Pruppacher, H.R., and Klett, J.D. Microphysics of clouds and precipitation. 2nd ed. Dordrecht Heidelberg London New York: Springer (2010).
[16] Karunakaran, G., Suriyaprabha, R., Rajendran, V., and Kannan, N. Effect of contact angle, zeta potential and particles size on the in vitro studies of Al_2_O_3_ and SiO_2_ nanoparticles. *IET Nanobiotechnol.* 9(1), 27-34 (2015). <https://doi.org/10.1049/iet-nbt.2013.0067>.
[17] Vlahakis, J.G. and Barduhn, A.J. Growth-rate of an ice crystal in flowing water and salt-solutions. *AICHE J.* 20, 581-591 (1974). <https://doi.org/10.1002/aic.690200320>.
[18] Anderson, D.M., Worster, M.G., Davis, S.H. The case for a dynamic contact angle in containerless solidification. *J. Cryst. Growth* 163(3), 329-338 (1996). <https://doi.org/10.1016/0022-0248(95)00970-1>.
[19] Shao, K.K., Song, M.J., Zhang, X. and Zhang, L. Growth and distribution characteristics of trapped air bubbles in ice slices. *Phys. Fluids* 35, 113319 (2023). https://doi.org/10.1063/5.0175106.
[20] Shao, K.K., Song, M.J., Shen, J., Zhang, X. and Pekař, L. Experimental study on the distribution and growth characteristics of trapped air bubbles in ice slices at different freezing temperatures. *Appl. Therm. Eng.* 244, 122600 (2024). https://doi.org/10.1016/j.applthermaleng.2024.122600.
[21] Miao, Y., Zhao, Y. and Gao, M. Characteristics of a freezing nanosuspension drop in two different schemes. *Appl. Phys. Lett.* 120, 091602 (2022). https://doi.org/10.1063/5.0084094.
[22] Zhao, Y., Yang, C. and Cheng, P. Freezing of a nanofluid droplet: From a pointy tip to flat plateau. *Appl. Phys. Lett.* 118, 141602 (2021). https://doi.org/10.1063/5.0044935.
[23] Jiang, Y.P., Zhao, Y.G., Zhang, H. and Cheng, P. Three-dimensional shaping strategy via solidifying polygonal nanofluid drops. *Cell Rep. Phys. Sci.* 5(4), 101904 (2024). https://doi.org/10.1016/j.xcrp.2024.101904.
[24] Yu, W. and Choi, S.U.S. The role of interfacial layers in the enhanced thermal conductivity of nanofluids: a renovated Maxwell model. *J. Nanopart. Res.* 5, 167-171 (2003). <https://doi.org/10.1023/A:1024438603801>.
[25] Maxwell, J.C. Treatise on electricity and magnetism. 2nd ed. Oxford University Press, Cambridge, UK (1904).
[26] Prasher, P. Thermal conductivity of nanoscale colloidal solutions (nanofluids). *Phys. Rev. Lett.* 94, 025901 (2005). <https://doi.org/10.1103/PhysRevLett.94.025901>.
[27] Trivedi, M.D. and Johansen, C.T. Forced convective heat transfer in Al_2_O_3_-air nanoaerosol. AIAA Propulsion and Energy Forum, 2015.
[28] Murshed, S.M.S., Leong, K.C. and Yang, C. Enhanced thermal conductivity of TiO_2_-water based nanofluids. *Int. J. Therm. Sci.* 44(4), 367-373 (2005). https://doi.org/10.1016/j.ijthermalsci.2004.12.005.
[29] Molgaard, J. and Smeltzer, W.W. Thermal conductivity of magnetite and hematite. *J. Appl. Phys.* 42, 3644-3647 (1971). https://doi.org/10.1063/1.1660785.
[30] Cahill, D.G., and Pohl, R.O. Thermal conductivity of amorphous solids above the plateau. *Phys. Rev. B* 35, 4067-4073 (1987). https://doi.org/10.1103/PhysRevB.35.4067.
[31] Zahhaf, O., D’Ambrogio, G., Le, M.Q., Coativy, G., Grasland, F., Cottinet, P.J. and Capsal, J.F. Dielectrophoretic alignment of Al_2_O_3_/PDMS composites: enhancement of thermal and dielectric properties through structural sedimentation analysis. *Mater. Design* 211, 110134 (2021). https://doi.org/10.1016/j.matdes.2021.110134.
[32] Pillai, S.C., Periyat, P., George, R., McCormack, D.E., Seery, M.K., Hayden, H., Colreavy, J., Corr, D. and Hinder, S.J. Synthesis of high-temperature stable anatase TiO_2_ photocatalyst. *J. Phys. Chem. C* 111, 4, 1605-1611 (2007). https://doi.org/10.1021/jp065933h.
[33] Sepúlveda, R., Plunk, A.A. and Dunand, D.C. Microstructure of Fe_2_O_3_ scaffolds created by freeze-casting and sintering. *Mater. Lett.* 2015, 142, 56-59. <https://doi.org/10.1016/j.matlet.2014.11.155>.
[34] Götze, J. Mineralogy and mineral chemistry of quartz: A review. *Mineral. Mag.* 85, 639-664 (2021). https://doi.org/10.1180/mgm.2021.72.
[35] Carte, A.E. Air bubbles in ice. *Proc. Phys. Soc.* 77, 757-768 (1961). <https://doi.org/10.1088/0370-1328/77/3/327>.
[36] Epstein, P.S. and Plesset, M.S. On the stability of gas bubbles in liquid-gas solutions. *J. Chem. Phys.* 18(11), 1505-1509 (1950). https://doi.org/10.1063/1.1747520.
[37] Yoshimura, K., Inada, T., and Koyama, S. Growth of spherical and cylindrical oxygen bubbles at an ice-water interface. *Cryst. Growth Des.* 8(7), 2108-2115 (2008). https://doi.org/10.1021/cg070251k.
[38] Dean, R.B. The formation of bubbles. *J. Appl. Phys.* 15, 446-451 (1944). <https://doi.org/10.1063/1.1707453>.
[39] Cole, R., Papazian, J.M., and Wilcox, W.R. Bubble departure radii at solidification interfaces. *Int. J. Heat Mass Transfer* 23, 219-224 (1980). <https://doi.org/10.1016/0017-9310(80)90198-2>.
[40] Stauffer, B., and Berner, W. CO_2_ in natural ice. *J. Glaciol.* 21, 291-300 (1978). <https://doi.org/10.3189/S0022143000033487>.
[41] Bari, S.A., and Hallett, J. Nucleation and growth of bubbles at an ice-water interface. *J. Glaciol.* 13, 489-520 (1974). <https://doi.org/10.3189/S0022143000023248>.
[42] Shao, K.K., Song, M.J., Cai, B.H., Zhang, L., Pekar, L., and Zhang, X. Effects of environmental temperature and trapped air bubbles on the mechanical properties of ice cubes. *ACS Appl. Mater. Interfaces* 16(46), 63482-63494 (2024). <https://doi.org/10.1021/acsami.4c12227>.
[43] Petrovic, J.J. Review Mechanical properties of ice and snow. *J. Mater. Sci.* 38, 1-6 (2003). <https://doi.org/10.1023/A:1021134128038>.
[44] Masoumi, N., Sohrabi, N. and Behzadmehr, A. A new model for calculating the effective viscosity of nanofluids. *J. Phys. D Appl. Phys.* 42(5), 055501 (2009). https://doi.org/10.1088/0022-3727/42/5/055501.
